# Human Endometrial Extracellular Matrix Hydrogel Facilitated Endometrial Mesenchymal Stem Cells for Endometrial Regeneration

**DOI:** 10.1002/adhm.202501767

**Published:** 2025-09-21

**Authors:** Jingwen Xu, Philip C N Chiu, Ernest H Y Ng, Sentao Hu, Zi Ye, Liaobing Xin, Lie Ma, Songying Zhang, William S B Yeung, Rachel W S Chan

**Affiliations:** ^1^ Department of Obstetrics and Gynaecology School of Clinical Medicine LKS Faculty of Medicine The University of Hong Kong Hong Kong SAR China; ^2^ Shenzhen Key Laboratory of Fertility Regulation The University of Hong Kong Shenzhen Hospital Shenzhen Guangdong China; ^3^ MOE Key Laboratory of Macromolecular Synthesis and Functionalization Department of Polymer Science and Engineering Zhejiang University Hangzhou Zhejiang 310058 China; ^4^ Key Laboratory of Reproductive Dysfunction Management of Zhejiang Province Assisted Reproduction Unit Department of Obstetrics and Gynecology Sir Run Run Shaw Hospital Zhejiang University School of Medicine Hangzhou Zhejiang 310016 China

**Keywords:** decellularization, endometrium, extracellular matrix, hydrogel, mesenchymal stem cell

## Abstract

The extracellular matrix (ECM) constantly remodels to tailor a temporal spatial specific environment for the residing cells to respond to physiological or pathological stimuli. Endometrial mesenchymal stem cells (eMSC) are excellent therapeutic candidates for treating endometrial problems. In‐depth investigation of the native niche to understand the regulatory mechanisms of the stem cells will enable greater translational potentials in regenerating the thin or damaged endometrium. To understand the ECM niche of eMSC, endometrial ECM from full thickness human endometrial tissues at different menstrual phases are preserved by tissue decellularization and then transformed into hydrogel material (EndoGel). EndoGel exhibits excellent compatibility with eMSC by enhancing the expansion of eMSC in vitro and facilitating the therapeutic regenerative effect in vivo evidenced by the improved fertility outcome. Comparative study of the proliferative to secretory phase EndoGel reveals unique matrisome at specific phase of the human menstrual cycle. The post‐regenerated endometrium shows distinct transcriptomic profile when transplanted with different menstrual phase EndoGel, suggesting the regulatory effect of the tissue matrix is menstrual phase specific. This is the first study comparing the endometrial matrix from specific human menstrual cycle and exploring its therapeutic potentials as a supportive biomaterial for eMSC to enhance endometrial regeneration.

## Graphical abstract

Adequate thickness of the uterine lining is crucial for women with thin or damaged endometrium to achieve pregnancies. Endometrial mesenchymal stem cells (eMSC) can facilitate the cyclical repair and regeneration of the human endometrium. In this study, the human endometrium derived extracellular matrix hydrogel supports the growth of eMSC promoting tissue regeneration.

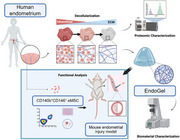

## 1 Introduction

Human endometrium is the mucosal lining of the uterus that serves as the interface for embryo implantation and undergoes cyclic changes during women's reproductive age.^[^ ^1^ ^]^ Although the distinct regenerative capacity of the endometrium can sufficiently respond to physiological or acute pathological stress, severe or repetitive damage can result to irreversible changes. In vitro fertilization (IVF) can overcome infertility caused by various factors in many patients. However, once the endometrium is severely damaged, the effect of IVF will be hindered as the “final destination” of embryo has been impaired.^[^ ^2^ ^]^

Human endometrial mesenchymal stem cells (eMSC) localized in perivascular regions of the endometrium and are responsible for tissue regeneration activities. In vitro characterization of this cell population fulfills the MSC criteria suggested by the International Society for Cell Therapy (ISCT): (1) adhesion to plastic surfaces under culture conditions, (2) expression of cell surface markers CD44, CD90, CD105, and CD73, (3) lack of expression of hematopoietic markers, and (4) ability to differentiate into osteoblasts, chondroblasts, and adipocytes in vitro.^[^ ^3^ ^]^ Evidenced by their therapeutic effect in regenerating the mouse injured endometrium in vivo, eMSC holds great potential as a cell source for treating endometrial infertility.^[^ ^4^ ^]^

Primary eMSC are a rare population of cells, constituting only ≈1–4% of endometrial stromal cells located in the basalis and functionalis.^[^ ^3^, ^5^, ^6^ ^]^ To achieve sufficient cell number for translational applications MSC must be expanded. However, prolong in vitro culturing can result in spontaneous differentiation, replicative senescence, and cell death, which hinder the potential for therapeutic usage.^[^ ^7^ ^]^ Endometrial MSC cultured with the presence of niche‐related components can better maintain their stem cells properties in vitro.^[^ ^8^, ^9^ ^]^ Optimizing the in vitro expansion strategy as well as the cell delivery platform for maximum in vivo therapeutic effect of eMSC demands better understanding of the regulatory factors of eMSC. In this study, we aim to engineer a native microenvironment biomaterial to facilitate the therapeutic use of eMSC.

Extracellular matrix (ECM) is a key niche factor that regulates stem cell functions. The proteomic composition and biomechanical properties of ECM are tissue‐specific and undergoes constant remodeling in response to environmental stimuli.^[^ ^10^ ^]^ The dynamic features of the human endometrium show distinct biological and structural features at each phase of the menstrual cycle, resulting in a phase‐specific ECM profile.^[^ ^11^ ^]^ The exploration into the temporal spatial specific ECM was powered by the decellularization techniques.^[^ ^12^ ^]^ The decellularized tissue can be transformed into hydrogel, and these native matrix derivatives can promote regeneration of damaged tissues.^[^ ^13^ ^]^ In conjugation with cellular therapy, hydrogels exhibit great therapeutic potential by enhancing cell functions.^[^ ^14^ ^]^

To date, most of the published studies of endometrial ECM and hydrogel derived from endometrial ECM are from tissue of non‐human sources such as porcine and bovine.^[^ ^15^, ^16^ ^]^ Despite these studies provide valuable knowledge on the endometrial matrix components and hydrogel modelling, the subsequent work has limited physiological relevancy since the human menstrual cycle is distinct from that of other animals. Furthermore, these published studies focus predominately on the biomaterial biocompatibility to bulk cell types in the endometrium or endometrial epithelial organoids. Little is known about the impact of biomaterials on the endometrial stem cells.

In this study, human endometrial tissues from proliferative or secretory phase were transformed into hydrogel (EndoGel) and their biomechanical and proteomic properties were compared. The functional roles of the EndoGel on eMSC were then evaluated using the mouse endometrial injury model.

## 2 Results

### 2.1 Characterization of Human Endometrium Derived EndoMatrix and EndoGel

Full thickness endometrial tissues were obtained from women undergoing hysterectomy for benign gynecological conditions. Tissue decellularization was achieved by incubation with decellularization buffer containing equal volume of 0.25% Triton X‐100 and 0.25% sodium deoxycholate (SDC). The menstrual phase of the sample was recorded and the resulting decellularized endometrial tissue is referred as EndoMatrix. The EndoMatrix was then transformed into hydrogel designated as EndoGel.

**Figure 1:**
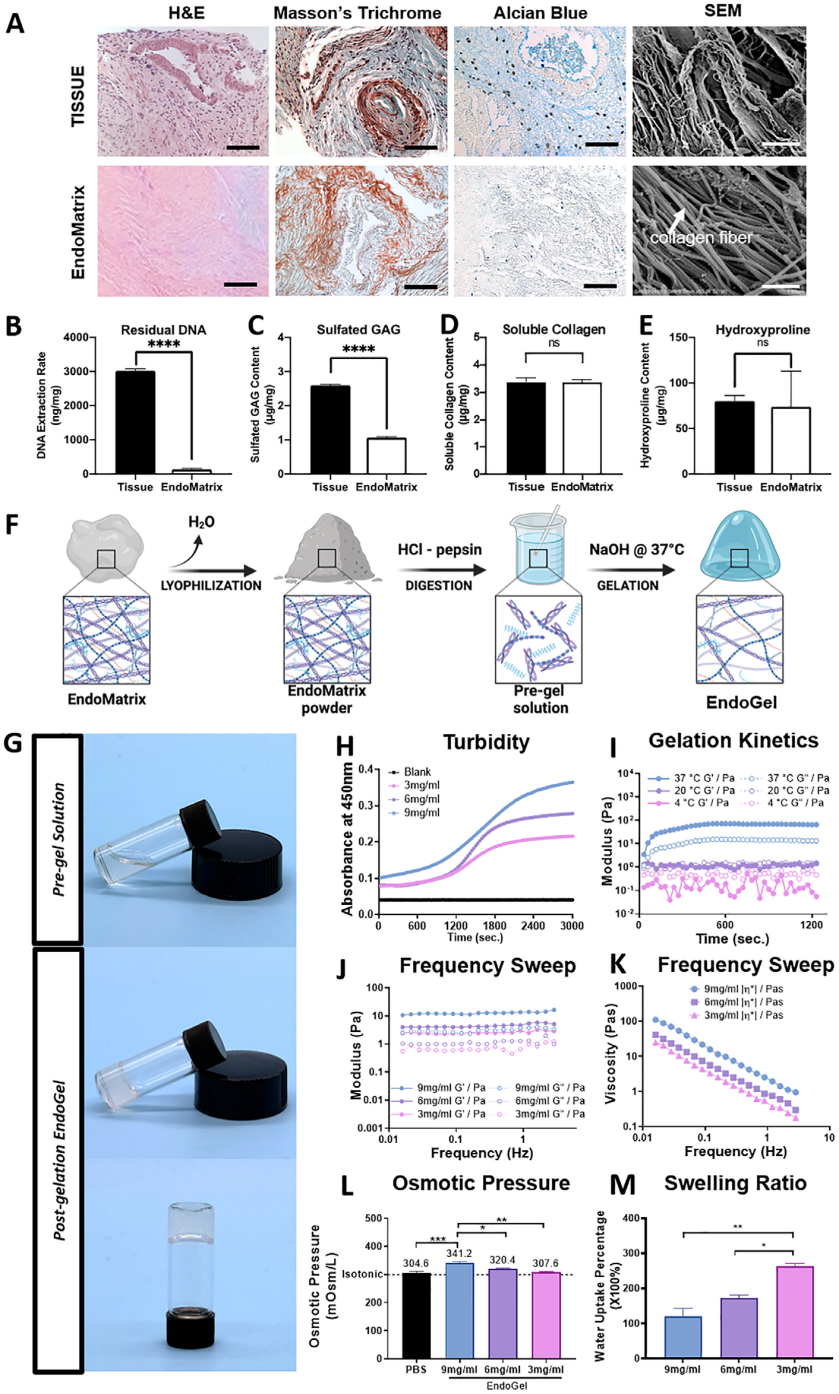
Characterization of EndoMatrix and EndoGel derived from human endometrial tissues. A) Representative images of H&E, Masson's Trichrome, Alcian Blue, and SEM imaging of human endometrial tissue before/after decellularization. Scale bars = 50 µm for H&E, Masson's Trichrome, Alcian Blue. Scale bars = 500 nm for SEM. B) DNA content of human endometrial tissue before/after decellularization, n = 3. C–E) Quantification of sulfated GAG, soluble collagen, and hydroxyproline of human endometrial tissue before/after decellularization, n = 3. F) Schematic illustration of the manufacturing of EndoGel (created in BioRender). G) Images of 6 mg mL^−1^ EndoGel before/after gelation. H) Turbidimetric assessment for gelation kinetics of EndoGel at 3, 6, and 9 mg mL^−1^. I) Rheological assessment for gelation kinetics of 6 mg/ml EndoGel at 4, 20, and 37 °C. J, K) Frequency sweep rheological assessment of EndoGel. L) Osmotic pressure n = 3 and M) swelling ratio of EndoGel, n = 3. Proliferative and secretory samples were pooled for the evaluation of EndoMatrix and EndoGel. Statistical significance assessed by an unpaired two‐tailed *t*‐test between two groups or by the ordinary one‐way ANOVA with Tukey's multiple comparison test between multiple groups. Data shown as mean ± SEM. * *p* < 0.05, **p < 0.01, ***p < 0.001, ****p < 0.0001. (Abbreviations: H&E, hematoxylin & eosin; SEM, scanning electron microscope; GAG, glycosaminoglycan).

To evaluate the decellularization efficiency, histological staining, DNA quantification, and ECM quantification were performed. Using H&E stain, the dark purple stained nuclei were depicted in the original tissue (**Figure** **1**A). Absence of such hematoxylin staining in the EndoMatrix indicated thorough removal of cellular nuclei by decellularization. The two key components of ECM, namely collagen and glycosaminoglycans (GAGs), were visualized by Masson's Trichrome staining and Alcian Blue staining respectively. The bright blue color in the Masson's Trichrome staining indicated preservation of the collagen, while the blue color in the Alcian Blue staining suggested retention of the GAGs (Figure 1A). Scanning electron microscope (SEM) images were used to assess the microstructures in the native tissue and its decellularized EndoMatrix counterpart. The endometrial tissue revealed a more complex structure with cellular components, while the decellularized tissue comprised of fibrous structures with intact D‐bands of collagen (Figure 1A). Consistent with the staining results, the DNA quantity was significantly reduced to a minimum level after decellularization (Figure 1B). Quantification of the major ECM component showed that the sulfated GAG (sGAG) level decreased after decellularization (Figure 1C). However, both the total collagen (quantified as hydroxyproline) and soluble collagen were well preserved after decellularization (Figure 1D,E). Together, these results demonstrated that the current decellularization protocol is efficient at removing the cellular content while preserving the key ECM profile of human endometrium.

The transformation of EndoMatrix to EndoGel was achieved by lyophilization, acid‐enzyme digestion, and neutralization (Figure 1F). The initial digestion was performed at 12 mg mL^−1^ to allow room for further dilution and neutralization. Three different concentrations of pre‐gel solution were obtained by dilution with PBS (3, 6, and 9 mg mL^−1^). Gelation of the pre‐gel solution was achieved at 37 °C with a pH of 7.5 (Figure 1G–I). The gelation kinetics of EndoGel was determined using turbidimetric (Figure 1H) and rheological (Figure 1I) assessments.

The turbidity of EndoGel increased during the incubation at 37 °C, with a segment of rapid increase at ≈1200 to 1800 s. The final turbidity was associated with the concentration of the pre‐gel solution. A longer turbidity increasing time was needed for the 9 mg mL^−1^ EndoGel when compared to the 3 and 6 mg mL^−1^ EndoGel (Figure 1H; Figure S1A, Supporting Information). The rheological properties of the 6 mg mL^−1^ pre‐gel including the storage modulus (G′), loss modulus (G″), and viscosity (η) were time‐elapsed monitored with an oscillatory rheometer at 37 °C (gelation temperature), 20 °C (preparation temperature), and 4 °C (short‐term preservation temperature). At 4 °C, the storage modulus was constant and less than the loss modulus, indicating a liquid status. Both the storage modulus and the loss modulus increased when the pre‐gel solution was at 20 °C, which remained constant over the observation period, indicating that the gelation was not triggered under 20 °C. When the pre‐gel solution was loaded at 37 °C, a rapid increase of the storage modulus and the loss modulus was observed, which was stabilized within 500 s with a final status of the storage modulus larger than the loss modulus, indicating a solid status (Figure 1I).

The post‐gelation EndoGel was assessed for biomechanical properties with the oscillatory rheometer frequency sweep test (Figure 1J,K). Linear viscoelasticity was observed with frequencies ranging from 0.01 to 1 Hz for the EndoGel at different concentrations, indicating that the EndoGel was a biomechanically stable material. The storage modulus of the EndoGel was at a level of less than 100 Pa, highlighting the softness of the material (Figure 1J). The shear viscosity measurement decreased with increase of the oscillatory frequency. This shear thinning effect implied the flexibility of the EndoGel after solidification (Figure 1K).

For cells to survive when in contact with the EndoGel, it is essential to keep the osmotic pressure of the EndoGel at an isotonic level. The pH neutralization process of the pre‐gel solution introduced additional salt to the buffer that caused changes of the osmotic pressure (Figure 1L). The osmometric assessment of various concentrations of EndoGel ranged between 300–350 mOsm L^−1^. There was a decrease of osmotic pressure along with the EndoGel concentration, with 9, 6, and 3 mg mL^−1^ EndoGel showing an osmotic pressure of 341.2 (± 5.398), 320.4 (± 3.265), and 307.6 (± 4.130) mOsm L^−1^ respectively.

As a key feature of hydrogel material, the water uptake ability (indicated by swelling ratio) of EndoGel was assessed (Figure 1M). Intuitively, the water containing ability increased when the EndoGel concentration decreased, with 9, 6, and 3 mg mL^−1^ EndoGel uptaking the amount of water as large as 119.4 (± 24.02), 172.4 (± 8.349), and 262.8 (± 8.992) × 100% of the dry weight of the gel, allowing cell signaling and metabolites to be buffered. The EndoMatrix and EndoGel used for the above analysis were from proliferative and secretory phase samples. For subsequent experiments 6mg/ml EndoGel was selected as it displayed balanced gelation rate, stiffness, osmotic pressure and swelling ratio when compared to the other two concentrations.

### 2.2 Proteomic Characterization of EndoMatrix and EndoGel from Proliferative and Secretory Phase

In response to the cyclical signals, the human endometrial tissue produces phase‐specific ECM across the menstrual cycle. The protein constitution of the EndoMatrix from the proliferative (n = 3) and secretory (n = 3) phase was characterized and compared from a proteomic perspective. The inter‐donor variability needs to be considered when interpreting the data reported since limited patient samples were tested.

**Figure 2:**
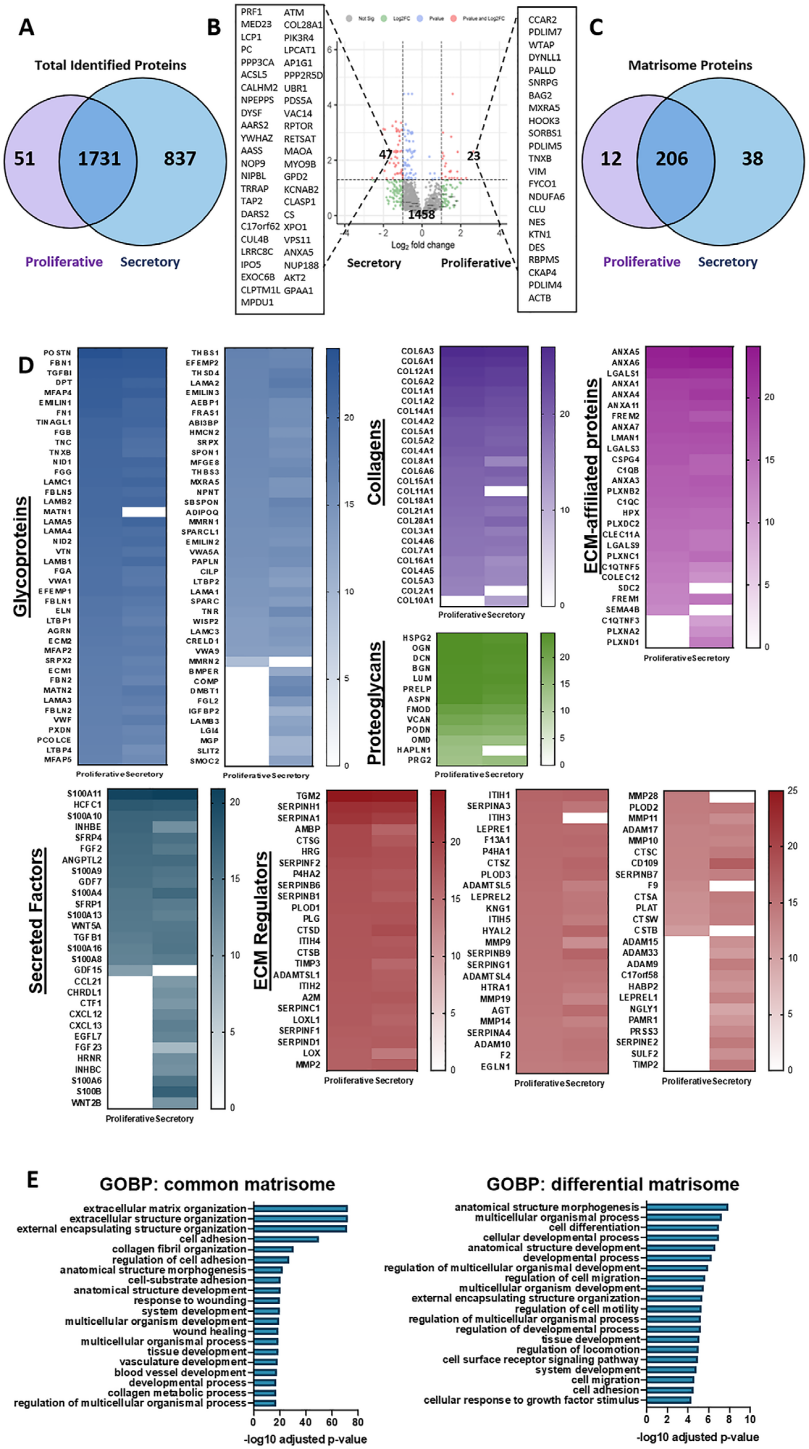
Proteomic comparative analysis of EndoMatrix from proliferative and secretory phase. A) Venn diagram of proteins identified from proliferative (n = 3) and secretory (n = 3) phase EndoMatrix. B) Volcano plot of differentially expressed proteins identified from proliferative and secretory phase EndoMatrix. C) Venn diagram of matrisome proteins identified from proliferative and secretory phase EndoMatrix. D) Heatmap representing the profile of matrisome proteins from proliferative and secretory phase EndoMatrix. E) Gene Ontology enrichment analysis of common and differential matrisome proteins from proliferative and secretory phase EndoMatrix. (Abbreviations: ECM, extracellular matrix; GOBP: Gene Ontology Biological Process).

A total of 1782 and 2568 proteins were identified from the proliferative and secretory phase samples respectively, with 1731 proteins commonly expressed in both phases (Figure **2**A). A differentially expressed protein analysis revealed that 23 proteins were significantly more expressed at the proliferative phase, while 47 proteins were significantly more expressed at the secretory phase. A total of 1458 proteins were similarly expressed by both phases (Figure 2B).

The identified proteins from the samples were mapped to the matrisome database, which was constructed to identify all the proteins constituting the ECM or participating in the matrix bioactivity in physiological or pathological conditions.^[^ ^17^ ^]^ It serves as an inventory of ECM proteins and their associated modifiers for comparative study in ECM research.^[^ ^10^ ^]^ Matrisome proteins from the proliferative phase (218/1782, 12.23%) and the secretory phase (244/2568, 9.50%) were identified, whereby 206 were commonly found in both groups (Figure 2C). To visualize the abundance of individual matrisome protein in the two groups, a matrisome map (Figure 2D) was constructed. The secretory phase sample contained a wider range of matrisome proteins, which mainly belong to the category of glycoproteins, secreted factors, and ECM regulators when compared to proliferative phase. The common matrisome and differential matrisome proteins were processed for functional clustering analysis. As expected, the common matrisome proteins formed biological process clusters of extracellular matrix organization, extracellular structure organization, external encapsulating structure organization, cell adhesion, and collagen fibril organization. Meanwhile, the differential matrisome enriched for functions such as anatomical structure morphogenesis, multicellular organismal process, cell differentiation, cellular developmental process, and anatomical structure development, implying the differential effects from the phase‐specific protein profile (Figure 2E).

**Figure 3:**
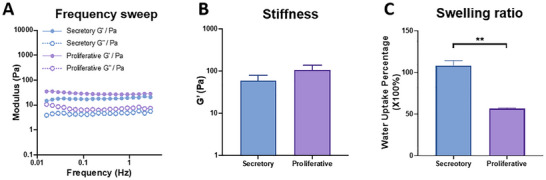
Biochemical assessment comparing EndoGel from proliferative and secretory phase. A) Frequency sweep rheological assessment of proliferative and secretory phase EndoGel. B) Storage modulus (stiffness) of proliferative and secretory phase EndoGel, n = 4. C) Water uptake ability of proliferative and secretory phase EndoGel, n = 3. Statistical significance assessed by an unpaired two‐tailed *t*‐test between two groups. ** *p* < 0.01. Data shown as mean ± SEM.

Next, the biomechanical properties after transformation of the EndoMatrix to EndoGel was studied. Proliferative and secretory phase EndoGel successfully formed stable gel as indicated by linear viscoelasticity (**Figure** **3**A). When assessing the stiffness as represented by the storage modulus (G’), there was no difference between the proliferative (107.2 ± 31.3) and secretory phase (59.47 ± 20.4) EndoGel (Figure 3B). Interestingly, the secretory EndoGel showed almost twice the water containing ability (107.9 ± 6.3) when compared to proliferative EndoGel (56.42 ± 0.94, *p* < 0.05, Figure 3C). These biomechanical assessments demonstrated the secretory EndoGel was relatively softer and absorbed more water. The increased abundance of glycoproteins and ECM regulatory factors identified in the secretory EndoGel may influence the physical and biological properties of the hydrogel.

### 2.3 Supportive Role of EndoGel on eMSC Expansion In Vitro

The cytotoxicity of EndoGel on eMSC was assessed with the extraction medium toxicity assay (Supplementary Figure S1B, Supporting Information). The 24‐hour extraction medium of EndoGel at 3, 6, and 9 mg mL^−1^ was used to culture eMSC. The cell survival remained similar at the different time points (24, 72, 120, and 168 h). Cell growth was also similar across all concentrations, indicating that the EndoGel was not toxic to eMSC in vitro.

The compatibility of eMSC embedded with EndoGel was evaluated using live/dead cell imaging at different time points. The green fluorescence signals (Calcein‐AM^+^) were indicative of live cells, and the red fluorescence (EthD‐1^+^) labelled dead cells. Over the 5‐day observation period, the bright green signals were observed uniformly in the EndoGel, with limited red signals detected (Supplementary Figure S1C, Supporting Information). Generally, the viability (proportion of cells alive among all cells) of eMSC embedded in EndoGel remained more than 90% from day 1 (94.05 ± 1.4%), day 3 (94.36 ± 1.7%), to day 5 (93.81 ± 1.9%) (Supplementary Figure S1D, Supporting Information), suggesting that EndoGel provides a viable environment for cell survival.

**Figure 4:**
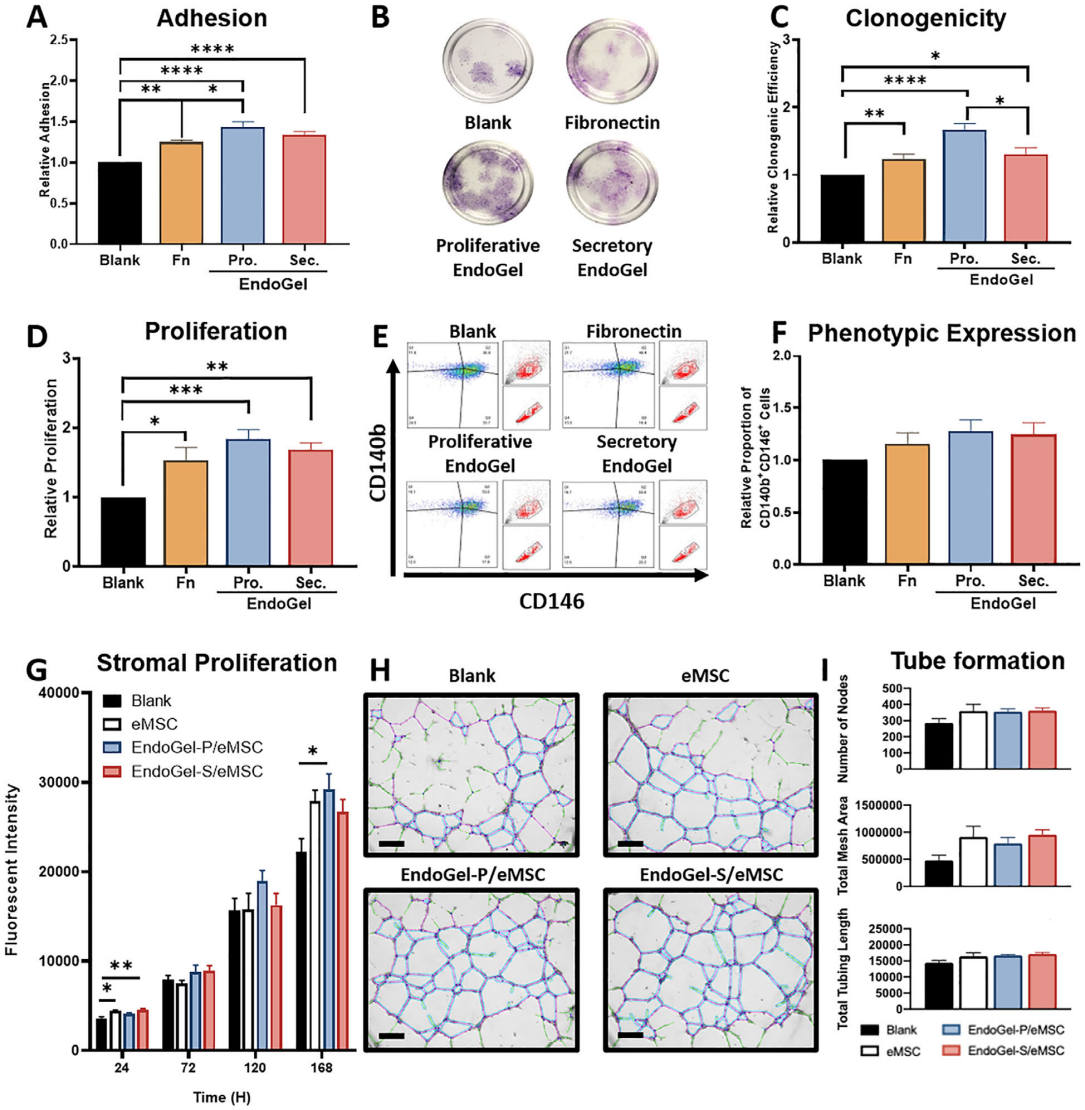
Supportive role of EndoGel on eMSC expansion in vitro. A) The relative adhesion activity of eMSC, n = 5. B) Representative crystal violet staining of colonies formed by eMSC. C) The relative colony formation activity of eMSC, n = 6. D) The relative proliferation activity of eMSC, n = 7. E) Representative scatter plot of eMSC surface marker CD140b and CD146. F) The relative CD140b^+^CD146^+^ cell proportion of eMSC, n = 6. G) Proliferation of primary endometrial stromal cells treated with secretome of eMSC with/without EndoGel, n = 5. H) Representative post‐process tube formation images of HUVEC treated with secretome of eMSC with/without EndoGel. Scale bar = 300 µm. I) Quantification of tubes formed by HUVEC treated with secretome of eMSC with/without EndoGel, n = 5. EndoGels at 6 mg mL^−1^ were used. Statistical significance between multiple groups was assessed by the ordinary one‐way ANOVA with Tukey's multiple comparison test for parametric data or Kruskal‐Wallis with Dunn's multiple comparison test for non‐parametric data. Data shown as mean ± SEM. *p < 0.05, **p < 0.01, ***p < 0.001, ***p < 0.0001. (Abbreviations: Fn, fibronectin; Pro, proliferative; Sec, secretory; HUVEC, human umbilical vein endothelial cells).

To assess whether the EndoGel could support the in vitro expansion of eMSC, various functional assays were performed. EndoGel coated culture plates were compared with the tissue culture polystyrene (TCPS) control and fibronectin coated plates. Fibronectin is the standard culture coating to support eMSC growth.^[^ ^18^ ^]^ Compared to the TCPS control, eMSC seeded on fibronectin (1.25 ± 0.03 fold, *p* < 0.01), proliferative EndoGel (1.43 ± 0.06 fold, *p* < 0.0001), and secretory EndoGel (1.34 ± 0.06 fold, *p* < 0.0001) displayed significantly better relative adhesion efficiency (Figure **4**A). The proliferative EndoGel also displayed better adhesion ability than fibronectin (*p* < 0.05).

Next, the ability of eMSC to form clones was determined (Figure 4B,C). Endometrial MSC seeded on fibronectin (1.23 ±0.07, *p* < 0.05), proliferative (1.67 ± 0.10, *p* < 0.0001) and secretory (1.31 ± 0.010, *p* < 0.05) phase EndoGel formed significantly more clones than the TCPS control. Meanwhile eMSC seeded on EndoGel provided significant improvement in clonogenic activity, showing a larger effect in the proliferative phase when compared to the secretory phase treated group (*p* < 0.05).

The proliferative activity of eMSC was assessed over a 5‐day culture period (Figure 4D). After being cultured for 5 days, the eMSC seeded on fibronectin, proliferative and secretory EndoGel showed significant enhancement on the proliferation activity indicated by the resulting cell quantity, with a relative proliferation efficiency of 1.53 ± 0.18 (*p* < 0.05), 1.83 ± 0.13 (*p* < 0.001), and 1.68 ± 0.11 (*p* < 0.01) fold to the eMSC seeded on the TCPS control.

The preservation of stem cell markers is an indicator of the stemness maintenance during in vitro expansion. Culture in the presence of the proliferative EndoGel (1.28 ± 0.11 fold) and the secretory EndoGel (1.24 ± 0.11 fold) remained the same as the TCPS control (Figure 4E,F).

The paracrine function of eMSC embedded with/without EndoGel was explored to test how the secretome of eMSC influence the functions of other cells (Figure 4G–I). Stromal proliferation was evaluated first. Secretome of eMSC (*p* < 0.05) and from secretory EndoGel/ eMSC (*p* < 0.01) promoted the proliferation of endometrial stromal cells within the first 24 h (Figure 4G). The enhancing effect was detected at 168 h for the eMSC group and the proliferative EndoGel/eMSC group (*p* < 0.05). Next, the pro‐angiogenic effect of the secretome of eMSC was assessed using the tube formation assay. The secretome of eMSC with or without EndoGel did not show any statistically significant difference in comparison with the blank (Figure 4H,I).

Overall, these findings indicate that the EndoGel can support the growth and expansion of eMSC while promoting the paracrine function of eMSC that can benefits the endometrial regeneration. Furthermore, the EndoGel from the proliferation phase demonstrated a superior effect in most of the studied parameters.

### 2.4 EndoGel Improved the Endometrial Regeneration in a Mouse Endometrial Damage Model

**Figure 5:**
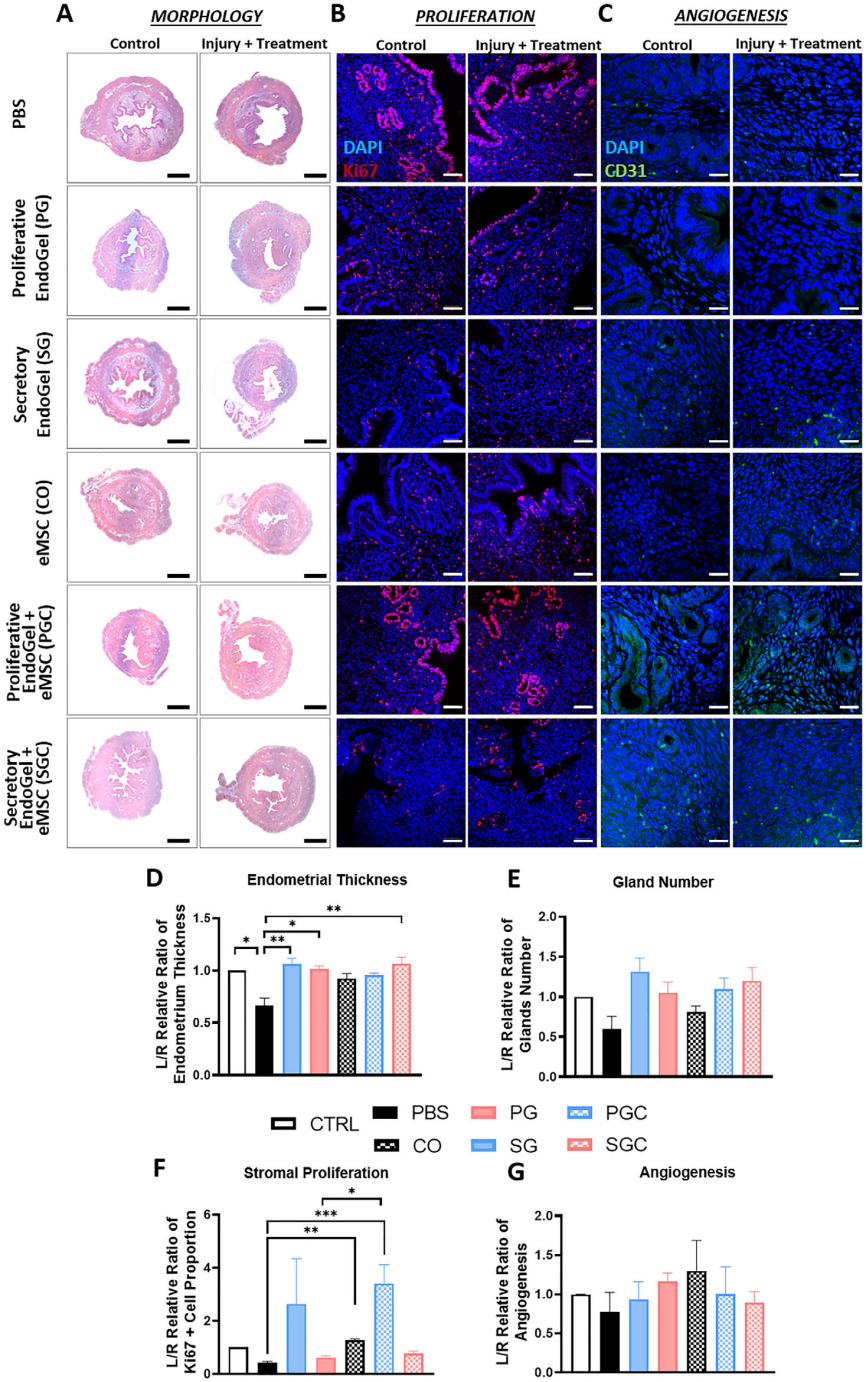
Damaged mouse endometrium regenerated after treatment with EndoGel and eMSC. Representative cross‐sectional A) H&E and B,C) immunofluorescent staining images of mouse uterine horn 7 days post‐operation. (Blue: DAPI, Red: Ki67, Green: CD31). Scale bars: 100 µm. D–G) Quantification of the normalized endometrial thickness, glands number, stromal proliferation, and angiogenesis of mouse uterine horn treated with EndoGel and eMSC at day 7 post‐operation, n = 6. The damaged uterine horn (L) was normalized against the undamaged control uterine horn (R) of the same animal. Statistical significance between multiple groups was assessed by Kruskal‐Wallis with Dunn's multiple comparison test. Data shown as mean ± SEM. *p < 0.05, **p < 0.01, ***p < 0.001. (Abbreviations: CTRL, undamaged control; PBS, damaged with PBS administered (no treatment); PG, damaged with proliferative EndoGel; SG, damaged with secretory EndoGel; CO, damaged with eMSC cell only; PGC, damaged with proliferative EndoGel/eMSC; SGC, secretory EndoGel/eMSC).

An electrocoagulation mouse endometrial damage model has demonstrated the therapeutic effect of eMSC on endometrial regeneration.^[^ ^4^ ^]^ The surgical procedure was performed at diestrus phase to minimize the variation of the estrus cycle between animals and allow damage to reach the deeper region of the endometrium. The left uterine horn of each mouse was injured with or without treatment, while the right uterine horn was unoperated as the control. Addition of PBS served as the natural repair control. Mouse endometrium at post‐operative day 7 was evaluated with histological and immunofluorescent staining. The regenerated mouse endometrium for morphological assessment was visualized using H&E staining (Figure **5**A). As expected upon injury the endometrial thickness deceased significantly when compared to control (*p* < 0.01, Figure 5A,D). Both the proliferative EndoGel (PG: 1.06 ± 0.06, *p* < 0.01) and the secretory EndoGel (SG: 1.02 ± 0.03, *p* < 0.05) greatly accelerated the endometrial thickness restoration when compared to the PBS control (PBS: 0.66 ± 0.08, Figure 5A,D).

In the presence of eMSC, only together with secretory EndoGel (SGC: 1.01 ± 0.03) significantly improved the endometrial thickness when compared to PBS (CO: 0.93 ± 0.05, *p* < 0.01, Figure 5A,D). For restoration of endometrial glands, an increasing trend was detected for the PGC and SGC treatment group. However due to large variation, it was not statistically significant (Figure 5E). Fluorescent labeled eMSC were detected within the mouse uterine cavity at post‐operative day 7 (Supplementary Figure S3B, Supporting Information).

Regeneration of the endometrium requires replenishment of the endometrial stromal cells. When treated with EndoGel without eMSC, no prominent effect was observed for the PG (2.64 ± 1.71) and SG group (0.61 ± 0.07, Figure 5B,F). Interestingly, the presence of eMSC contributed toward promoting stromal cell proliferation. All the treatment groups with eMSC resulted in a high proportion of proliferating stromal cells when compared to PBS (PBS: 0.42 ± 0.06; CO:1.27 ± 0.05, *p* < 0.01; PGC: 3.41 ± 0.71, *p* < 0.0001; SGC: 0.78 ± 0.08, *p* < 0.05, Figure 5B,F).

Angiogenesis is another key event in endometrial regeneration. The angiogenic activity was quantified by staining CD31 in the mouse endometrium (Figure 5C). No major differences were observed among the damaged endometrium in PBS (0.77 ± 0.25), PG (0.93 ± 0.23), SG (1.16 ± 0.11, Figure 5C,G) groups. Angiogenic activity was improved when the damaged mouse endometrium was treated with eMSC in comparison to the PBS group and to their EndoGel only counterparts. However, all groups failed to reach any significance due to the large variation (CO:1.30 ± 0.39; PGC: 1.00 ± 0.35; SGC: 0.90 ± 0.14, Figure 5C,G).

### 2.5 Endometrial MSC Administered with EndoGel Significantly Improved the Fertility of Mice with Damaged Endometrium

**Figure 6:**
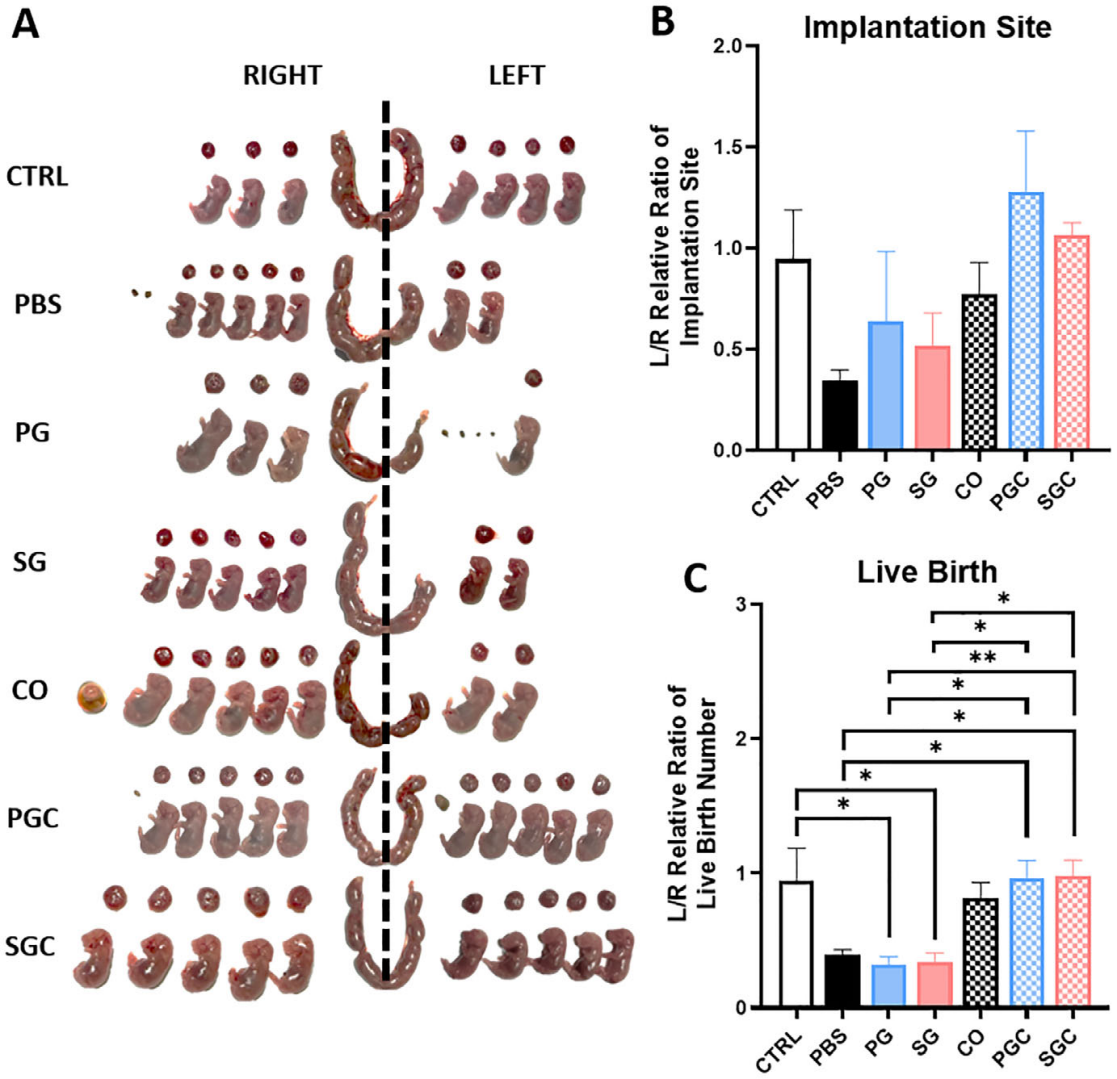
Fertility test of mouse endometrium regenerated after treatment with EndoGel and eMSC. A) Representative images of mouse uterine horns, placenta, and fetus at gestation day 18.5. Quantification of the normalized B) implantation site number and C) live birth number of mouse uterine horn treated with EndoGel and eMSC, n = 4–6. The damaged uterine horn (L) was normalized against the undamaged control uterine horn (R) of the same animal. Statistical significance between multiple groups was assessed by the ordinary one‐way ANOVA with Tukey's multiple comparison test. Data shown as mean ± SEM. * *p* < 0.05, ** *p* < 0.01. (Abbreviations: CTRL, undamaged control; PBS, damaged with PBS administered (no treatment); PG, damaged with proliferative EndoGel; SG, damaged with secretory EndoGel; CO, damaged with eMSC cell only; PGC, damaged with proliferative EndoGel/eMSC; SGC, damaged with secretory EndoGel/eMSC).

Besides the morphological and molecular assessments, fertility test was performed to functionally assess whether the post‐regenerated endometrium support embryo implantation and fetal growth. At gestation day 18.5, the pregnant mice were harvested to assess the pregnancy outcome (Figure **6**A). Based on the gestational sac status, the pregnancy outcome was categorized into (1) viable birth (normal gestational sac with viable fetus, confirmed by yolk sac pulsation at harvest), (2) non‐viable birth (gestational sac with unviable, restricted, or malformed fetus), and (3) resorption (resorbed implantation material, without gestational sac formation). The pregnancy outcome is summarized in Table **1**.

**Table 1:** Pregnancy outcome of the different treatment groups.

Tx	Viable birth number		Non‐viable birth number		Resorption number		Average fetus weight Mean (± SD)		Average placenta weight Mean (± SD)	
	L	R	L	R	L	R	L	R	L	R
CTRL	9	10	0	0	0	0	NA	NA	NA	NA
PBS	8	21	0	0	0	4	1.00 (± 0.11)	1.02 (± 0.2)	0.09 (± 0.02)	0.09 (± 0.01)
PG	6	19	0	1	4	0	1.09 (± 0.14)	1.163 (± 0.12)	0.099 (± 0.065)	0.10 (± 0.01)
SG	5	16	0	0	1	0	1.05 (± 0.13)	1.00 (± 0.17)	0.091 (± 0.01)	0.10 (± 0.02)
CO	11	15	0	2	0	0	0.95 (± 0.09)	1.03 (± 0.11)	0.10 (± 0.02)	0.10 (± 0.012)
PGC	16	17	0	0	5	1	0.98 (± 0.11)	1.11 (± 0.09)	0.09 (± 0.02)	0.09 (± 0.01)
SGC	15	15	0	0	1	0	0.78 (± 0.13)	0.88 (± 0.16)	0.10 (± 0.01)	0.11 (± 0.02)

Abbreviations: Tx, treatment; L, left; R, right; CTRL, undamaged control; PBS, damaged with PBS only; PG, damaged with proliferative EndoGel; SG, damaged with secretory EndoGel; CO, damaged with eMSC cell only; PGC, damaged with proliferative EndoGel / eMSC cell; SGC, damaged with secretory EndoGel / eMSC cell; SD, standard deviation; NA, not available.

With the undamaged right‐side uteri served as internal control, the L/R ratio of implantation sites (Figure 6B) and number of viable births (Figure 6C) provided indication on the relative fertility restoration. The PG treated mice (relative implantation ratio: 0.64 ± 0.35 fold; relative viable birth ratio: 0.33 ± 0.05 fold) and SG treated mice (relative implantation ratio: 0.51 ± 0.16 fold; relative viable birth ratio: 0.34 ± 0.07 fold) showed minimum improvement in implantation rate and viable birth rate when compared to the PBS group (relative implantation ratio: 0.34 ± 0.06 fold; relative viable birth ratio: 0.39 ± 0.04 fold).

Interestingly, the proliferative PGC (relative implantation ratio: 1.28 ± 0.30 fold; relative viable birth ratio: 0.96 ± 0.13 fold, *p* < 0.05), and SGC (relative implantation ratio: 1.06 ± 0.06 fold, *p* < 0.05; relative viable birth ratio: 0.98 ± 0.12 fold, *p* < 0.05) revealed a significant improvement on the birth rate when compared to the PBS group (relative implantation ratio: 0.34 ± 0.06 fold; relative viable birth ratio: 0.39 ± 0.04 fold). The presence of eMSC with the EndoGel resulted in better viable births for proliferative (PGC vs PG, *p* < 0.05) and secretory (SGC vs SC, *p* < 0.05) phase when compare to the EndoGel alone. These results suggest the EndoGel may enhance the therapeutic effect of eMSC in vivo. The quality of fetus and placenta was assessed by recording the fetus and placenta weight at the time of harvest. Only weights of viable births were included. The recorded weights were averaged for each uterine horn then normalized against the readings from the right‐side control. Overall, no significant differences were observed amongst the different treatment groups (Supplementary Figure S3C,D, Supporting Information). Expression of the receptivity markers integrin alpha v (Supplementary Figure S4A, Supporting Information) and HOXD10 (Supplementary Figure S5A, Supporting Information) showed stronger expression in groups with treatment compared to the PBS control.

### 2.6 The Transcriptomic Profile of Post‐Regenerated Mouse Endometrium was Dependent of the Menstrual Phase of EndoGel

**Figure 7:**
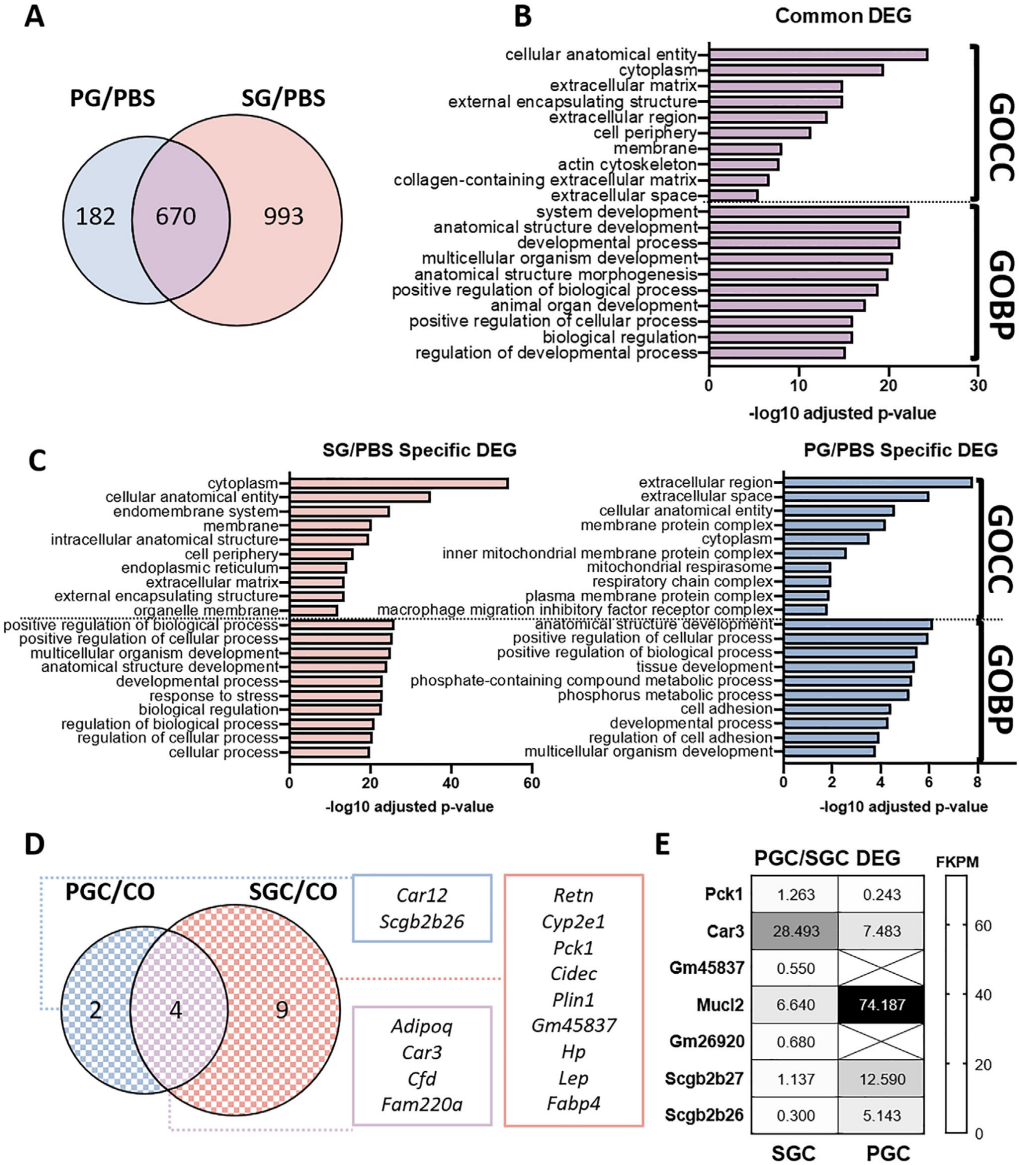
Transcriptomic analysis of the post‐regenerated mouse uterine tissue. A) Venn diagram of DEGs of post‐regenerated mouse endometrial tissues treated with EndoGel (n = 3 / experimental group). Gene Ontology enrichment analysis of B) common and C) specific DEGs. D) Venn diagram of DEGs of post‐regenerated mouse endometrial tissues treated with EndoGel/eMSC. E) Heatmap of DEGs between post‐regenerated mouse endometrial tissues treated with SGC and PGC. (Abbreviations: DEG, differentially expressed genes; GOCC, gene ontology cellular component; GOBP, gene ontology biological process; PBS, damage only; PG, proliferative EndoGel; SG, secretory EndoGel; CO, cell only; PGC, proliferative EndoGel/eMSC; SGC, secretory EndoGel/eMSC; FKPM, fragments per kilobase of transcript per million mapped reads).

Differentially expressed gene analysis was performed for bulk RNA sequencing dataset of the regenerated mice uterine horns to compare the therapeutic effect of EndoGel from the proliferative and secretory phase at post‐operative day 7 (Figure **7**). When assessing the regenerated endometrium after transplantation of EndoGel, 852 DEGs were identified from PG and 1663 DEGs were found from SG in comparison to the PBS group. A total of 670 genes were commonly regulated in both treatment groups (Figure 7A).

These commonly regulated genes were clustered with GO enrichment analysis (Figure 7B). For cellular component GO terms, the top enriched clusters include cellular anatomical entity, cytoplasm, extracellular matrix, external encapsulating structure, and extracellular region. For biological process terms, top enriched clusters include system development, anatomical structure development, developmental process, multicellular organism development, and anatomical structure morphogenesis. These results highlighted common therapeutic effects brought by the EndoGel with a focus on structural reconstruction in tissue regeneration.

The uniquely regulated genes by PG and SG were also compared and revealed a differential pattern of the functional clusters (Figure 7C). For cellular component terms, the endometrium regenerated with SG was mainly related to cytoplasm and cellular anatomical entity, while the PG treated tissue was regulated for extracellular region and space. For biological process terms, tissue development, several metabolic process, and cell adhesion were highly enriched in the PG treated endometrium, whereas response to stress and regulation process was enriched in the SG treated endometrium.

Next, the comparison between PGC and SGC treated mouse endometrium was made with CO as the control. As indicated by the mouse regenerated endometrium assessment and fertility test, the groups treated with eMSC showed significant restoration in morphology and function at post‐operative day 7. Thus, limited DEG was identified when comparing these groups. Only 6 and 13 genes were differentially regulated for PGC *vs* CO and SGC *vs* CO comparatively, with 4 common genes detected (Figure 7D). *Pck1, Car3, Gm45837, Mucl2, Gm26920, Scgb2b27, and Scgb2b26* were the 7 genes that were differentially expressed by the PGC and SGC group when directly comparing the sequencing dataset of the two groups (Figure 7E). Overall, the transcriptomic analysis of the regenerated mouse endometrium revealed a menstrual phase‐specific therapeutic effect of the EndoGel without eMSC. When EndoGel was administered together with eMSC, the regenerated uterine horns showed minimal differences.

## 3 Discussion

Endometrial stem cells in the functionalis and basalis play vital roles in regenerating the endometrium.^[^ ^19^ ^]^ Regulation and expansion of eMSC require culture conditions that can recapitulate the complexity of the endometrial niche. The present study investigating the human endometrial ECM in a menstrual phase specific manner has unveiled key regulatory factors in the matrisome profile that can be used in designing a biomaterial for eMSC delivery. In this study, the EndoGel model served two purposes. As a study model, the EndoGel preserved the protein complexes for comparative analysis of the ECM in the proliferative and secretory phase, potentiating the identification of important factors for eMSC regulation. As a cell delivery platform, the biomechanical properties of the EndoGel were characterized for its cytocompatibility and therapeutic effects with eMSC for tissue regeneration to assess its potential in translational clinical use. Thus, the EndoGel is a valuable tool for studying the dynamic aspects of endometrial regeneration.

### 3.1 The Manufacturing of Extracellular Matrix Hydrogel from Human Endometrium

To manufacture EndoGel with desired cytocompatibility and physiological relevance to the original tissue, each step including decellularization, dissolution, and gelation requires optimization. The current strategies on decellularization of endometrium involves ionic detergents, non‐ionic detergents, or a combination of both.^[^ ^20^ ^]^ To decellularize bovine endometrium, Jamaluddin et al. studied three decellularization protocols by assessing the biochemical, proteomic, and biomechanical properties of the resulting hydrogels.^[^ ^15^ ^]^ Compared to the SDS based protocols, SDC based decellularization showed better preservation of the native matrisome and demonstrated greater support for organoid growth. However, the current protocol resulted in a considerable loss of GAGs when comparing the tissue before/after decellularization. Similar observation has been reported.^[^ ^21^ ^]^ Loss of GAGs can result in a less physiological matrix profile. Further optimization of the decellularization protocol will be needed in the future. A recent published review appeals to reach consensus among researchers in the field of decellularization to establish assessment criteria standards for decellularized biomaterial evaluation.^[^ ^22^ ^]^ Such consensus will benefit future study designs and empower the decellularization based biomaterial for clinical translation. Moreover, the loss of GAGs in ECM hydrogel can impact various types of applications. In vitro, reduction of GAG levels can reduce tissue stiffness, impair biomineralization and alter tissue structure.^[^ ^23^, ^24^ ^]^ In vivo, the loss of GAGs can delay growth of new tissue and integration with the host tissue. A less supportive microenvironment will impede the hydrogel's ability to form functional tissue and compromise tissue regeneration.^[^ ^25^, ^26^ ^]^

Dissolution of the decellularized matrix involves acid‐enzymatic digestion that breaks the solid matrix into small particles in a liquid suspension format (bio‐ink). The digestion duration and acid strength need to be balanced to achieve thorough digestion while not over‐digesting the matrix. While pepsin is commonly used across studies as the proper enzyme for digestion, the acid type and concentration has been under discussion. Zhao et al. investigated the influence of different acidic solution types on hydrogel formation.^[^ ^15^ ^]^ When compared to acetic acid, hydrochloric acid showed increased digestion efficiency, resulting in a softer hydrogel with lower stiffness which was favored by the embedded cells. More importantly, the osmotic pressure of acetic acid digested matrix cannot be lowered to physiological state while the hydrochloric acid digested matrix after PBS adjustment can reach isotonic osmotic pressure, suggesting hydrochloric acid is a better option for cell embedding hydrogels. In the present study, acid‐enzymatic digestion with 0.1 M hydrochloric acid and 0.1 mg/mg pepsin resulted in a soft, isotonic, cyto‐compatible hydrogel that is optimal for modelling.

From a biomaterial perspective, the application potential of EndoGel depends greatly on the performance and characteristics at the designated application scenario. Characterization of the EndoGel was carried out to assess the morphology, gelation kinetics, biomechanical stability and strength, swelling ratio, osmotic pressure, cytotoxicity, and cytocompatibility. Evidenced by the gelation kinetics, EndoGel remains as a liquid at 4 and 20 °C. It can then quickly form as a gel when put at 37 °C, indicating that temporary storage and manipulation will not result in unwanted gelation. When EndoGel is injected into the uterine cavity, the biomaterial can quickly solidify to prevent leakage.

The solid form of EndoGel displayed excellent stability as indicated by the rheological analysis where linear viscoelasticity was observed. The stiffness of human endometrial tissue is 400–600 kPa, while hydrogels derived porcine or bovine endometrium range between 10 to 400 kPa.^[^ ^15^, ^16^, ^27^ ^]^ A hydrogel with higher stiffness can provide a pro‐inflammatory environment.^[^ ^28^ ^]^ Lower stiffness hydrogel as presented in this study creates an anti‐inflammatory environment, suitable for tissue restoration. Moreover, the stiffness gradient created at the hydrogel‐tissue interface can promote migration of MSC toward the tissue via durotaxis. Excessively low stiffness risks hydrogel instability. Orthopedic regenerative studies have shown biomaterial stiffness can regulate the fate of MSC.^[^ ^29^, ^30^ ^]^ To enhance the therapeutic functions of eMSC, optimization of the hydrogel stiffness is needed in the future. Establishing the physiological stiffness profile of human endometrium throughout the menstrual cycle would facilitate this process and improve understanding of the native endometrial biomechanics.

Capturing a great amount of water and being isotonic, the EndoGel created an environment where the residing cells can easily metabolize and function. The optimized protocol established that the EndoGel material is a stable biocompatible isotonic soft biomaterial which will be useful as a research model and therapeutic cell delivery vehicle.

### 3.2 Menstrual Phase Specificity of the Endometrial ECM

The endometrium undergoes constant remodeling in response to the shifting sex steroids, resulting in variation of the ECM constitution and architecture across the menstrual cycle. The cyclic shedding of the endometrium occurs only in higher order primates and cannot be depicted in studies using bovine or porcine tissues. Endometrial tissue obtained from reproductive aged women allows the study of the matrix environment at specific phases of the menstrual cycle.

The endometrial matrix at specific menstrual phase has been previously investigated. Certain matrix components, such as collagen III and V, are present across the menstrual cycle, yet the morphology and organization changes from a condensed structure in the proliferative phase to structural channels between cells in the secretory phase.^[^ ^11^ ^]^ The matrix components including type IV collagen, laminin, and heparan sulphate proteoglycans had limited expression and are localized to mainly around the blood vessel walls and glandular basement membrane during proliferative phase, while the expression of these components increased by mid and late secretory phase. Although the main structural protein – collagen was relatively similar across the menstrual cycle. Findings from the proteome / matrisome analysis revealed certain glycoproteins, ECM regulators and secreted factors were detected in secretory phase in comparison to the proliferative phase. For example, the abundance of ADAM members, SERPIN 2, TIMP‐2 detected from secretory phase samples demonstrate their role in ECM breakdown during endometrial remodeling. Decidualization of the endometrial tissue in secretory phase resulted in higher tissue hydration, with a less compact and expanded extracellular space.^[^ ^11^ ^]^ Whether certain proteomes contribute toward the softness and water containing features of the secretory phase EndoGel will need further investigation.

### 3.3 EndoGel Supported eMSC Functions

The matrix hydrogel effect on different types of endometrial cells have been reported. Human endometrial stromal cells were more hormonal responsive and underwent decidualization when seeded on porcine derived ECM hydrogel when compared to collagen.^[^ ^16^ ^]^ Embryos attachment also enhanced when Ishikawa cells in monolayer were cultured on plates coated with endometrial ECM hydrogel.^[^ ^16^ ^]^ Matrigel as a tumor derived matrix complex is widely used for organoid formation and culture. Jamaluddin et al. proposed that human endometrial organoids require a more physiological culture condition that was not ill‐defined for better modelling function.^[^ ^15^ ^]^ Their study investigating the function and proteome of the organoids cultured in bovine endometrial ECM hydrogel and Matrigel concluded that the ECM hydrogel was superior to Matrigel in providing a more native environment for healthy and natural growth of endometrial organoids. To our knowledge, no study has assessed the compatibility and the promoting effect of matrix hydrogel on stem cells from the endometrium, specially eMSC. Hence, the present study pioneered at establishing the relationship between tissue matrix hydrogel and MSC derived from the endometrial tissue.

EndoGel was cytocompatible at supporting the functions of eMSC, displaying no cytotoxicity and supported the functions of eMSC. Arising from the low stiffness and isotonic osmotic pressure, the biomechanical properties of the EndoGel favored extended cell growth. The live/dead staining showed eMSC had an overall survival rate over 90% across 5 days of culture. The high water‐containing ability of EndoGel also provided the buffer room required by cell metabolism. Both the proliferative and secretory phase EndoGel displayed stimulatory effects on eMSC with the proliferative EndoGel exhibiting greater effects. When assessing the paracrine actions of eMSC embedded in the proliferative EndoGel, a better promoting effect of the endometrial stromal growth was observed at the later timepoint. In the cycling endometrium, the proliferative phase is when rapid regrowth of the stroma occurs. Consistent with such a phenomenon, the proliferative phase matrix is hypothesized to provide a favorable niche for stem cells to rapidly replace the cell loss while maintaining its stemness. This hypothesis was further explored with the mouse endometrial injury model.

### 3.4 EndoGel Facilitated the Regenerative Therapeutic Effects of eMSC

Since hydrogel derived from other non‐reproductive organ failed to improve the morphology and function of mouse injured endometrium, it was clear that the endometrium or full thickness uterus derived ECM hydrogel display unique therapeutic activities at sites of injury.^[^ ^16^ ^]^ The same study also highlighted a differentially regulated gene profile of the regenerated uterus when treated with different layers of the uterus (endometrial specific layer vs full thickness), highlighting a specific regulatory effect delicately dependent on the tissue origin.

Utilizing the electrocoagulation mouse damage endometrial model, the therapeutic effect brought by the EndoGel from proliferative and secretory phase with or without the eMSC were assessed. Consistent with other studies, EndoGel alone displayed considerable improvement in the morphological regeneration of the injured endometrium. However, a morphologically well‐regenerated endometrium is not a sufficient indicator of functional restoration. For fertility testing, EndoGel alone groups showed limited effect in restoring the number of viable births at the injured site. Inclusion of eMSC into the treatment improved the number of viable births observed, highlighting the importance of eMSC for functional restoration. Together with the in vitro experimental findings, the EndoGel enhanced the therapeutic effects of eMSC by providing a more suitable niche for the stem cells. The secreted factors and other constituting molecules of the EndoGel may also participate in creating a pro‐regenerative environment that works synergistically with eMSC.

When the injured endometrium was treated with the PG or PGC, a significant enhancement of the proliferating stromal cells was observed. The phase‐specific therapeutic effects were further confirmed by RNA‐seq analysis of the post‐regenerated mouse endometrium. Metabolic processes and tissue development enriched in the PG treatment group may have contributed toward the proliferation activity observed during regeneration. It has been reported carbonic anhydrases play important role in uterine endometrium by maintaining the pH and ion balance for fertilization.^[^ ^31^ ^]^ Our findings revealed *Car3* and *Car12* were differentially regulated between the PGC and SGC groups. In mouse endometrium, several CAR forms including CAR12 have been detected and play a role in reproduction.^[^ ^32^, ^33^ ^]^ The interaction of EndoGel with eMSC may mediate specific functional status to the regenerating endometrial cells in the mouse endometrium. However, only a limited number of DEGs were detected in the current study. Earlier collection timepoints may unveil the unique actions of phase specific EndoGel with eMSC during the active regeneration process. Future investigation toward the uniquely expressed proteins belonging to each menstrual phase and mechanical properties of the EndoGel may reveal the factors contributing to the observed phase‐specific therapeutic effect. Alternatively, artificially constructed biomaterial base on the understanding of the native phase‐specific endometrial tissue will be valuable for translational use in treating women with disordered endometrium.

### 3.5 Limitations and Future Studies

Although decellularized ECM hydrogel is commonly used as a research model, the exact mechanism of ECM hydrogel gelation remains unclear. Current opinion suggests it is potentially due to the abundant regulating factors (i.e. glycoproteins and proteoglycans) that under physiological condition can modulate the collagen fragments to self‐assemble (cross‐linking), thus go through the transformation from liquid to solid.^[^ ^16^ ^]^ Future endeavor is needed in understanding the modifiable factors in the hydrogel model for customization of the biomaterial with desired biomechanical properties by either modification or combination with other materials.

In the mouse endometrial injured model, treatments were transplanted at diestrus phase. Hormonal changes can affect the mechanical properties of hydrogels as well as the behavior of the encapsulated cells.^[^ ^34^ ^]^ Determining the optimal time of delivery may promote the EndoGel and eMSC to repair more efficiently. In addition, tracking EndoGel with a protein label can monitor the degradation, distribution and interaction of the hydrogel in the surrounding tissue during the study period. The lack of significant differences observed between the undamaged control group and the PBS group for gland numbers, stromal proliferation and angiogenesis suggest the thermal injury by electrocoagulation was not efficient at destructing the endometrial tissue. Heat‐based damage can be variable in particular for smaller sample size. In future studies, we will consider using chemical induced damage i.e. ethanol for a more complete disruption and disintegration of the endometrium.

The regulatory effect of the EndoGel on eMSC has shown menstrual phase specificity as indicated by both in vivo and in vitro results. Despite these functional observations, the stem cell‐matrix interaction mechanism remains unknown. Future characterization of specific integrin receptors and adhesion molecules on eMSC should be considered. Another important aspect is the in vivo host response. Since hydrogel and stem cells can regulate immune responses, the immunoregulatory effect will also need to be explored.

The interaction of human endometrial phase‐specific matrix on stem cells fate will enhance its potential as a therapeutic intervention for personalized regenerative medicine. However, the practical application of this approach is constrained by several limitations. Ethical standards and safety evaluations should be considered including the risks of allergic reactions and pathogen transmission. Concurrently, standardization of the manufacturing process and quality control will guarantee product consistency and efficacy.

## 4 Conclusion

To our knowledge, this is the first‐time different menstrual phase‐specified decellularized endometrial matrix transformed into hydrogel were compared. We successfully manufactured EndoGel – a soft, stable biomaterial and compatible with eMSC. EndoGel supporting expansion and therapeutic functions of eMSC revealed the positive regulatory role of tissue ECM on tissue derived stem cells. Our findings provide valuable insight toward the development of a multifunctional composite system to enhance endometrial regeneration.

## 5 Experimental Section

### Human Tissues

Full thickness endometrial tissues were obtained from women aged between 42–56 years who underwent abdominal hysterectomy due to benign conditions unrelated to the endometrium (n = 25). After the surgical procedure, a piece of the endometrial tissue (1cm × 1cm) was dissected from the uterus and collected in the collection medium composed of 1% FBS (Life Technologies, USA), 1% L‐glutamine (Life Technologies), and 1% penicillin‐streptomycin (Sigma‐Aldrich, USA) in DMEM/F‐12 (Sigma‐Aldrich). The inclusion criteria for this study included pre‐menopausal women with regular menstrual cycles and not on hormonal therapy for a minimum of 3 months. Endometrial samples were categorized into proliferative (n = 18) and secretory (n = 7) phase according to the histopathology reports (Supplementary Table S1, Supporting Information). A written consent was acquired from participants prior to their recruitment to this study and an ethical approval was obtained from the Institutional Review Board of The University of Hong Kong/Hospital Authority Hong Kong West Cluster (UW20‐465) and The Institutional Review Board of the University of Hong Kong Shenzhen Hospital ([2018]94).

### Decellularization

The decellularization protocol was modified as described.^[^ ^35^ ^]^ The endometrial tissue was detached from underlying myometrium using a surgical blade and cut into small pieces (5 mm^2^) for the decellularization procedure. Equal volume of 0.25% Triton X‐100 and 0.25% sodium deoxycholate (Thermo Fisher Scientific, USA) in dH_2_O was added for incubation. Pieces of endometrium were incubated with the decellularization buffer for 48 h with constant shaking (160 rpm) at 37 °C. The tissue was rinsed with fresh PBS daily for 3 days at 4 °C. Tissue was incubated with ribonuclease (RNase, 100 µg mL^−1^, Worthington Biochemical Corporation, USA) and DNase I (150 IU mL^−1^, Worthington Biochemical Corporation) for 48 h with constant shaking (160 rpm) at 37 °C. After the final PBS wash for 24 h, the tissue was stored at −80 °C.

### Scanning Electron Microscope

Samples fixed with 2% glutaraldehyde buffer overnight at 4 °C and dehydrated in gradient ethanol series. The samples were dried with liquid carbon dioxide before being attached to an aluminum stub for gold‐sputter coating. Images were captured with a LEO 1530 FEG SEM (Zeiss, Germany) or Hitachi S‐3400N variable pressure Scanning Electron Microscope (Hitachi, Japan) at Electron Microscope Unit, The University of Hong Kong.

### Histological Staining

Decellularized and the native endometrial tissue were fixed in 4% PFA overnight then dehydrated in 70% ethanol for paraffin embedding. Tissues embedded in paraffin blocks were sectioned (5 µm). For histological evaluation, hematoxylin and eosin (H&E) staining, Alcian Blue (AB) staining, and Masson's trichrome (MT) staining were performed. For H&E staining, slides were deparaffinized and rehydrated with xylene and gradient ethanol. Hematoxylin (Sigma‐Aldrich, USA) were stained for 45 s then washed twice with tap water for 5 min. After dehydration with graded ethanol, slides were counterstained with Eosin (Sigma‐Aldrich) for 45 s then went through ethanol absolute 5 min for three times. The slides were mounted with mounting medium and air dried. For AB staining, Alcian Blue powder (Sigma‐Aldrich) was dissolved in 3% acetic acid to a final concentration of 1% (w/v). The Alcian Blue solution was used to stain the rehydrated slides for 30 min then counterstained with nuclear fast red for 10 min. MT staining was performed by Department of Pathology, The University of Hong Kong.

### Residual RNA

Native and the decellularized endometrial tissues were sampled for DNA extraction using the DNA extraction kit (Tiangen Biotech, China). The concentration and total quantity of the extracted DNA was measured with Nanodrop 2000 spectrophotometer and normalized to the wet weight of the tissue chunk.

### Collagen and sGAG Quantification

The ECM content of native and decellularized endometrial tissues were quantified. The wet weight from 3 samples were recorded and processed for preparation according to each assay. The quantity of sulphated GAGs (sGAG) was measured using the Blyscan Kit (Biocolor, UK). The papain extraction reagent consists of 50 mL of sodium phosphate buffer (Na_2_HPO_4 –_ NaH_2_PO_4_, 0.2 m, pH 6.4), 400 mg sodium acetate, 200 mg Ethylenediaminetetraacetic acid (EDTA) disodium salt, 40 mg cysteine Hydrochloric Acid (HCl), and 5 mg papain (Huasheng, China) was freshly prepared. Samples (100 mg) were digested in 2 ml of papain extraction reagent at 65 °C overnight and the supernatant was collected after centrifugation. The samples together with the sGAG standards were processed for the dye labeling and releasing process. Absorbance was read at 595nm with the ELISA reader. The quantity of sGAG in each sample was calculated from the standard curve. Soluble collagen level was measured using the Sircol 2.0 Kit (Biocolor, UK) according to manufacturer's protocol. Samples (100 mg) were digested in 1 ml of 0.1 mg mL^−1^ pepsin/0.5 m acetic acid buffer at 4 °C overnight with gentle shaking. The supernatant was collected after centrifugation. The samples and collagen standards were measured for the soluble collagen content by dye labeling and releasing method described in manufacturer's protocol. Soluble collagen concentrations and total mass were calculated based on the standard curve. Hydroxyproline Assay Kit (Cayman Chemical, USA) was used to measure the total collagen content. Samples (100 mg) were homogenized in 1 ml H_2_O and hydrolyzed with equal volume of 10M Sodium Hydroxide (NaOH) at 120 °C for 1 h. The hydrolyzed cell lysate samples (20 µL) were dried by heating at 65 °C for 90 min. Hydroxyproline was oxidized (Reagent 1) to a pyrrole intermediate that reacts with Ehrlich's reagent (Reagent 2) to create a chromophore described in manufacturer's protocol and measured with absorbance at 595 nm. Hydroxyproline standard was included for calculating the standard curve.

### Preparation of EndoGel

The protocol for EndoGel was optimized from published studies.^[^ ^15^, ^27^ ^]^ In brief, EndoMatrix was finely minced in PBS then centrifuged. After removal of the supernatant, the pellet was kept at −20 °C for 24 h for solidification then lyophilized overnight in a freeze dryer. The resulting EndoMatrix dry powder was digested to a concentration of 12 mg mL^−1^ using 0.1 m HCl supplemented with pepsin (Sigma‐Aldrich) under constant shaking (160 rpm) for 3 days at room temperature. The digested samples were neutralized to pH 7 by 10m NaOH (1% the volume of HCl). A drop‐by‐drop fine‐tuning of pH was then achieved by 1m NaOH with frequent check of the pH. This pre‐gel solution was then osmotically balanced with 10X PBS and diluted with 1X PBS to a final concentration of 3, 6, and 9 mg mL^−1^ and stored at −20 °C. Characterization of EndoGel (turbidity, rheological, water uptake and osmometric analysis) were performed from proliferative and secretory phase pre‐gels at 6mg mL^−1^.

### Turbidity Analysis

The gelation kinetics observation and turbidity monitoring were achieved by spectrophotometry. Pre‐gel solution of EndoGel (100 µL) were pipetted into 96‐well plates. The absorbance at 450 nm was measured every 30 s for a total duration of 50 min at 37 °C. Samples were loaded in triplicates.

### Rheological Analysis

The rheological properties of pre‐gel and EndoGel was performed using the HAAKE Modular Advanced Rheometer System MARS 60 (Thermo Fisher Scientific) paired with a steel 20 mm parallel plate geometry. Temperature controlled time elapsed shear sweep tests were performed at 4, 20, and 37 °C to evaluate the gelation kinetics of 6mg/ml pre‐gels. The pre‐gel solution (600 µL) was loaded between the parallel plate geometries after the experimental setup stabilized. Storage (G′), loss (G″) modulus, and shear viscosities were recorded for 1500 s with an oscillatory frequency of 1 Hz and 1% strain. Oscillatory frequency sweep tests were performed with frequencies ranging from 0.01 to 10 Hz under a strain of 1%. Pre‐gel solution (400 µL) was incubated for 30 min at 37 °C in a 20 mm round mold to form the EndoGel, then detached and moved onto the parallel plate geometry after the platform temperature reached 37 °C. Shear viscosities, storage (G′) modulus, and loss (G″) modulus were recorded over the frequency range. To determine the storage modulus (G’) of the EndoGel, the mean of all readings within the linear viscoelastic range obtained from the oscillatory frequency sweep test were used.

### Water Uptake Capacity

EndoGel was prepared at 37 °C until stabilized then immersed in PBS until reach equilibrium. The weight of the gel was measured as W(g). The gel samples were then put in tubes for lyophilization overnight. The dry weight of the gel was recorded as W(d). The water uptake capacity (swelling ratio) of the gel was calculated as [W(g)‐W(d)]/W(d) * 100%. All weights were averaged from 5 readings.

### Osmometric Analysis

Osmotic pressure of pre‐gel solution and PBS control was tested with a freezing‐point osmometer OM807 (VOGEL MedTec, Germany) with a fixed freezing point of −6.2 °C. Pre‐gel solution was loaded in an Eppendorf tube for reading. Operation of the osmometer was based on the manufacturer's protocol.

### Proteomic Analysis

EndoMatrix from proliferative (n = 3) and secretory (n = 3) phase were analyzed for their proteome by LC‐MS with Label Free Quantification (LFQ) method pooled from multiple donors and analyzed in one batch. Sample processing and LC‐MS analysis were performed at the Proteomics and Metabolomics Core Facility, Centre of PanorOmic Sciences (CPOS), The University of Hong Kong. Tissue was lyophilized then suspended in the Urea lysis buffer (8 m Urea in 50 mm TEAB). Proteins were first extracted by bead beating using Precellys homogenization (Bertin technologies) and probe sonication (MSE Soniprep), followed by centrifugation (15 000 g) for 25 min at 4 °C. The supernatant fraction was collected for protein quantitation using the Bradford assay (Thermo Fischer Scientific). Protein digestion was performed using the filter aided sample preparation (FASP) strategy (Wisniewski et al., 2009). For FASP, 100 µg proteins from each sample were subjected to reduction and alkylation by 50 mm TCEP and 50 mm 2‐chloroacetamide, respectively. Protein digestion was performed by adding trypsin (Promega, 1:25 enzyme‐to‐substrate ratio) and incubated for 18 h at 37 °C. Subsequent tryptic peptides were Speedvac dried and desalted using the C18 StageTips for LC‐MS analysis. Eluted peptides were analyzed with a nanoelute UHPLC coupled to a Bruker timsTOF pro mass spectrometer. The peptide mixture was loaded onto an Aurora C18 UHPLC column (75 µm i.d. × 25cm length × 1.6 µm particle size, IonOpticks, Australia). Chromatographic separation was carried out using a linear gradient of 2–30% of buffer B (0.1% formic acid in acetonitrile) at a flow rate of 250 nL min^−1^ over 100 min. MS data was collected over a m/z range of 100 to 1700, and a MS/MS range of 100 to 1700. Raw mass spectrometry data was processed using the MaxQuant 1.6.14.0. The raw data was searched using the Andromeda algorithm against Human Swissprot FASTA database (May 2022) containing 20,361 entries, using settings as below: oxidized methionine (M), and acetylation (Protein N‐term) were selected as dynamic modifications, carbamidomethyl (C) as fixed modifications with a minimum peptide length of 7 amino acids. Confident proteins were identified using a target‐decoy approach with a reversed database, a strict false‐discovery rate 1% at peptide and PSM level. Proteins identified from both conditions were quantified using the peptide LFQ intensities and their ratio obtained were used for label free quantitation to calculate the fold change. Data visualization and statistical data analysis was performed by the Perseus software version 1.6.13.0. Identified proteins were categorized according to the phase of the menstrual phase to form the Venn diagram. Differentially expressed protein (DEP) analysis was performed and displayed as volcano plots. The list of identified proteins from proliferative or secretory phase was individually profiled with GO functional enrichment with g:Profiler.^[^ ^36^, ^37^ ^]^ The proteomic profile from LC‐MS result was further mapped to matrisome database for comparison of the proliferative and secretory matrisome.^[^ ^17^ ^]^ Venn diagram was constructed to visualize the belonging of the matrisome proteins. Heatmap of each subcategory of matrisome was individually constructed to compare the expression level of the protein in the proliferative and secretory phase samples. GO functional enrichment was performed with the g:Profiler for the commonly expressed matrisome protein as well as the differentially expressed matrisome proteins. Graphing of the proteome and matrisome related analysis was facilitated with the EVenn and SRplot platform.^[^ ^38^, ^39^ ^]^

### Isolation of Endometrial Stromal Cells

The procedure for isolation of endometrial stromal cells was conducted as described.^[^ ^6^ ^]^ Full thickness human endometrial tissues were detached from the myometrium with preservation of the basal layer. Endometrial tissue was finely chopped with surgical blades and the minced tissues were subjected to digestion using 0.3 mg/ml collagenase type III and 40 µg mL^−1^ deoxyribonuclease type I (DNase I, Worthington Biochemical Corporation) in PBS under constant shaking in a water bath set at 37 °C. The digestion stopped after 1 hour with addition of an equal volume of the collection medium. The suspension was filtered through a 40 µm sieves (BD Bioscience, USA) to separate the dispersed cells from the undigested tissue. The undigested tissue underwent a second round of digestion to maximize the harvest of cells. After completion of the second cycle of digestion, the cell suspensions were combined and centrifuged with Ficoll‐Paque (GE Healthcare, Sweden) to remove the red blood cells. To eliminate the leukocytes, the cell suspension was incubated with anti‐CD45 antibody coated Dynabeads (Invitrogen, USA) for 45 min at 4 °C. Epithelial cells were removed by anti‐CD326 antibody‐conjugated microbeads (Miltenyi Biotech, USA) for 30 min at 4 °C. The resulting cells were considered as freshly isolated endometrial stromal cells, which were seeded onto 10 cm dishes coated with fibronectin (1 mg mL^−1^, Gibco, USA) and cultured in culture medium (CM) composing of 10% FBS (Life Technologies), 1% L‐glutamine (Life Technologies), and 1% penicillin‐streptomycin (Sigma‐Aldrich) in DMEM/F‐12 (Sigma‐Aldrich) in a humidified carbon dioxide incubator at 37 °C. The CM was replenished every 3 days. Primary human endometrial stromal cells from passage 1 to 3 were used in this study.^[^ ^8^ ^]^

### Magnetic Bead Selection for eMSC

CD140b^+^CD146^+^ eMSC were purified from endometrial stromal cells by positive selection of surface markers with magnetic beads.^[^ ^6^ ^]^ When the stromal cells reached around 80% confluency, the cells were trypsinized and incubated with phycoerythrin (PE) conjugated anti‐CD140b antibody (R&D Systems, USA) for 45 min at 4 °C. The cell suspension was washed with 0.1% PBS/BSA followed by a 15 min incubation with anti‐mouse IgG magnetic microbeads (Miltenyi Biotech) at 4 °C. The cell suspension after column separation resulted in CD140b^+^ cells. The CD140b^+^ cells were cultured for 7 days on fibronectin coated plates at a density of 1 × 10^5^ cells per 10 cm plate to allow detachment of the magnetic beads during cell expansion. The expanded CD140b^+^ cells were then trypsinized and incubated with anti‐CD146 antibody coated microbeads (Miltenyi Biotech) together with blocking reagent for 15 min at 4 °C for the CD146 selection. The resulting CD140b^+^CD146^+^ cells were considered as eMSC and used for subsequent experiments (Supplementary Figure S2, Supporting Information). The eMSC was cultured under an environment of 5% carbon dioxide and 21% oxygen in a humidified incubator at 37 °C with regularly change of CM unless otherwise specified.

### Cytocompatibility of EndoGel with eMSC

EndoGel at concentrations of 3, 6 and 9 mg mL^−1^ were immersed in CM of eMSC for 24 h at 37 °C to obtain the extraction medium. The eMSC (500 cells) were cultured in the extraction medium for 1, 3, 5, and 7 days in 96‐well plate. Culture medium alone was used as control. At the harvesting time point, the cells were trypsinized and analyzed with the CyQUANT Cell Proliferation Kit (Thermo Fisher Scientific). The diluted cell lysis buffer was mixed with CyQUANT GR dye according to the manufacture's protocol. This mixture was then added into each well including the empty wells as blank control. The plates were put into a fluorescence microplate reader with excitation at 485 nm and emission at 520 nm. The reading of the fluorescence intensity after deducing the background reads from blank control were recorded as indicator of the DNA content. The viability of eMSC embedded in the EndoGel was assessed with a calcein‐AM and ethidium homodimer‐1 (EthD‐1) live/dead cell quantification kit (Invitrogen). Endometrial MSC (1 × 10^4^ cells) were suspended in 10 µL of 6 mg mL^−1^ EndoGel pre‐gel solution and allowed to solidify on a µ‐slide. After 30 min, the well was topped up with culture medium. After 1, 3, and 5 days, live/dead staining was performed according to manufacturer's protocol. Briefly, cells were washed with PBS before adding in 40 µL of the dye (final concentration of 2 µm for calcein‐AM and 4 µm for EthD‐1) and incubated for 30 min. Visualization of the fluorescently labelled cells was performed at the Imaging and Flow Cytometry Core, CPOS, The University of Hong Kong with a Carl Zeiss LSM 880 inverted confocal microscopes and analyzed with the Zeiss LSM Zen 2019 software (Carl Zeiss). The percentage of viable cells was calculated as (live cells / total cells) × 100%.

### Functional Assessments of eMSC

EndoGel coating model was used to evaluate the effect of EndoGel on eMSC behavior such as adhesion, proliferation, colony formation, and stem cell marker preservation. EndoGel (1 mg mL^−1^) diluted with DMEM/F‐12 was used to coat culture plates for 30 min and incubated at 37 °C for stabilization. Fibronectin (1 mg mL^−1^) was used as positive control. For adhesion, eMSC was seed at 3000 cells/ 96‐well plate and assessed after 2 h with CyQUANT Cell Proliferation Kit (Thermo‐Fischer Scientific). For proliferation, eMSC was seed at 1000 cells/96‐well plate and assessed after 7 days with CyQUANT Cell Proliferation Kit. For colony formation, eMSC were cultured at low density in 6‐well plates (300 cells per well) for 14 days to form colony. The plates were fixed with 10% formalin and stained with crystal violet. The number of colonies formed were counted for each well. For stem cell marker preservation, multicolor flow cytometry was applied to examine the phenotypical expression of eMSC under different treatment after 7 days in culture. Harvested cells were double stained with PE conjugated anti‐CD140b antibody (1:10, mouse IgG1, R&D Systems, #FAB1263P) and APC conjugated anti‐ CD146 (1:20, mouse IgG1, Biolegend USA, #361016) in 0.5% BSA/PBS at 4 °C in the dark for 45 min. The Fluorescent Minus One (FMO) control was included for each antibody. The fluorescence of labeled cells was detected with CytoFlex flow cytometer (Beckman Coulter, USA), and the obtained data were analyzed with FlowJo software (Tree Star Inc., Ashland, USA).

### Collection of eMSC Secretome

A 3D embedding transwell model was used to evaluate the paracrine effect of eMSC when cultured with/without EndoGel. Endometrial MSC (1 × 10^5^ cells) were seeded with/without EndoGel in 24‐well insert placed in a 24 well plate. The cells were allowed to stabilize for 30 min before adding 1 ml of CM. The lower chamber of the culture plate was supplemented with 2 mL of CM for secretome collection. After 3 days, the medium containing secretome in the lower chamber was collected. The regulatory effect of the secretome on endometrial stromal cells and endothelial cells was assessed. For stromal cell proliferation assay, endometrial stromal cells seed at 1000 cells/96‐well plate and assessed after 1, 3, 5, and 7 days with CyQUANT Cell Proliferation Kit described above. For endothelial cell tube formation, human umbilical vein endothelial cells (HUVEC, Lonza, Switzerland) were cultured on 10 cm plates coated with 0.2% gelatin in DMEM/F‐12K (Sigma‐Aldrich) medium with 10% FBS, 30 µg mL^−1^ endothelial cell growth supplements (ECGS, Sigma‐Aldrich), and 0.1 mg/ml heparin. HUVEC at passage 3–6 was used. To assess the angiogenesis activity of HUVEC cells, 15‐well µ‐Slides (IBIDI, Germany) were used for tube formation. The wells were coated with Matrigel (Corning) for 30 min in a 37 °C incubator for gelation, then HUVEC (10 000 cells) were seeded into each well in with/without eMSC secretome. After 14 h, the µ‐Slides were viewed under an Axioskop Phase Contrast Microscope (Zeiss, Germany) and image captured with the Tcapture software (Version 3.9, Tucsen Photonics, China). Image quantification was performed with the ImageJ software plug‐in Angiogenesis analyzer 2.0.

### Animal Holding Conditions

Mice were kept in cages within environmentally controlled rooms with light/dark cycle of 12h/12h with free access to water and laboratory diet (LabDiet, USA) unless otherwise specified for the experimental condition. The mice were purchased and kept in the Centre of Comparative Medicine Research at The University of Hong Kong. All experimental procedures performed were approved by the Committee on Use of Live Animals in Teaching and Research, The University of Hong Kong.

### Animal Study Design

Female Nonobese Diabetic/Severe Combined Immunodeficiency (NOD‐SCID) mice at 6‐week‐old were used for the mouse endometrial electrocoagulation damage model as described.^[^ ^4^ ^]^ Sample size was calculated with the Power & Animal Number Calculator provided by Center for Comparative Medicine Research, The University of Hong Kong. Vaginal smear was performed with PBS to confirm that the mice were at diestrus stage to eliminate the variation brought by the estrous cycle. After anesthesia with ketamine and xylazine intraperitoneally, a vertical incision was made at the left abdominal wall to expose the left uterine horn. A small incision was made at the uppermost part of the uterine tube to allow insertion of a monopolar electrode into the uterine lumen. The electrode pen was inserted into the lumen to induce damage. Electrocoagulation was performed with a power of 80 W for 10 s per direction and repeated for 8 uniformly distributed directions. After the damage was established, PBS was injected to flush the tissue fragments, and the treatment was administered immediately in accordance with the study design of each group. The left side uterine horn was damaged by electrocoagulation and then randomly assigned into one of the six treatment groups (n = 6 – 9/treatment group). (1) *PBS only (PBS)* – injection of PBS (20 µL) into the damaged uterine site, (2) *EndoGel‐P* (*PG)* – injection of proliferative phase EndoGel (20 µL) into the damaged uterine site, (3) *EndoGel‐S (SG)* – injection of secretory phase EndoGel (20 µL) into the damaged uterine site, (4) *eMSC only (CO)* – injection of 5 × 10^5^ eMSC resuspended in PBS (20 µL) into the damaged uterine site, (5) *EndoGel‐P/eMSC (PGC)* – injection of 5 × 10^5^ eMSC resuspended in proliferative phase EndoGel (20 µL) into the damaged uterine site, and (6) *EndoGel‐S/eMSC (SGC)* – injection of 5 × 10^5^ eMSC resuspended in secretory phase EndoGel (20 µL) into the damaged uterine site. The right uterine horn of each mouse was undamaged and served as control *(CTRL)*. EndoGel at 6mg/ml were used and the phase‐specific pre‐gels were pooled from multiple donors. On the day of surgery, pre‐gel aliquots were thawed on ice prior to the surgical procedure. For EndoGel + eMSC groups, the eMSC was resuspended in the thawed EndoGel pre‐gel solution. Some eMSC were incubated in PBS containing 2 mg/mL CM‐Dil (Thermo Fisher) at 37 °C for 5 min, followed by 15 min incubation at 4 °C. An elongated pipette tip was injected through the small incision on the left uterine horn to deliver the treatment. Endpoint of the animal experiment for morphometric and histological analysis was at post‐operation day 7 (Supplementary Figure S3A, Supporting Information).

For the post‐regeneration mouse uterine tissue RNA‐bulk sequencing analysis, both sides of the uterine horns were injured to minimize the inter‐animal variation. Mice were randomly allocated into one of the three experimental groups (n = 3). (1) LEFT side: *PBS only (PBS)* – injection of PBS (20 µL) into the damaged uterine site; RIGHT side: *eMSC only (CO)* – injection of 5 × 10^5^ eMSC resuspended in PBS (20 µL) into the damaged uterine site, (2) LEFT side: *EndoGel‐P (PG)* – injection of proliferative phase EndoGel (20 µL) into the damaged uterine site; RIGHT side: *EndoGel‐S (SG)* – injection of secretory phase EndoGel (20 µL) into the damaged uterine site, (3) LEFT side: *EndoGel‐P/eMSC (PGC)* – injection of 5 × 10^5^ eMSC resuspended in proliferative phase EndoGel (20 µL) into the damaged uterine site; RIGHT side: *EndoGel‐S/eMSC (SGC)* – injection of 5 × 10^5^ eMSC resuspended in secretory phase EndoGel (20 µL) into the damaged uterine site. At post‐operative day 7, the uterine tissue was collected and extracted for RNA sequencing analysis.

### Histological and Immunofluorescence Staining

Mouse uterine horns were fixed in 4% paraformaldehyde (PFA) overnight and stored in 70% ethanol and paraffin embedded. Paraffin sections (5 µm) were deparaffinized and rehydrated with xylene and graded ethanol and stained for H&E as described above. Endometrial thickness of embedded uterine horns was measured cross‐sectionally. The vertical distance from the luminal epithelium to the endometrial‐myometrial interface was calculated using algorithms from the Image‐Pro Plus software (Version 6.0, Media Cybernetics) from 10 sections of the same uterine horn then averaged as indicator of endometrial thickness. Gland numbers were manually counted from 10 sections and averaged for each uterine horn. For immunofluorescence staining, antigen retrieval was performed with antigen retrieval buffer (DAKO, Germany). The sections were blocked with 5% BSA/PBS before incubation with primary antibodies (rabbit anti‐Ki67, 1:800, Abcam UK, #ab16667; rat anti‐CD31, 1:100, BD Bioscience, USA, #550274; rabbit anti ‐integrin alpha v, 1:100, Abcam, #ab76609; rabbit anti‐HOXD10, 1:50, Abcam, #ab172865) diluted with 1% BSA/PBS overnight at 4 °C. After extensive washing with PBS, the sections were incubated with secondary antibody (Donkey anti‐Rabbit IgG‐Alexa Fluor 568, 1:200, Thermo Fisher Scientific, #A10042; Goat anti‐Rat IgG‐Alexa Fluor 488, 1:200, Thermo Fisher Scientific, #A11006) for 1 hour at room temperature. Washed with PBST (0.05% Tween‐20 in PBS before nuclear counterstaining with 4’,6‐diamidino‐2‐phenylindole (DAPI, 1:1000, Thermo Fisher Scientific) and mounted with a fluorescence mounting medium (DAKO). Images were captured by a Carl Zeiss LSM 880 inverted confocal microscope (Carl Zeiss, Germany) and analyzed with the Zeiss LSM Zen 2019 software (Carl Zeiss). Quantification of Ki67^+^ stromal cells were manually counted using the Image J software and divided by the total number of stromal cells per image to obtain the proportion of proliferating stromal cells. Quantification of CD31 was calculated as the CD31 positive signal fluorescence intensity divided by the DAPI positive area by ImageJ. The damaged uterine horn was normalized against the control uterine horn of the same animal.

### Fertility Test

At post‐operative day 7–10, female mice from different treatment groups (n = 4–6) were randomly assigned to mate with male NOD‐SCID mice. For the mating setup, 2 females were caged with 1 male overnight. Copulation plug detection was marked as 0.5 day of gestation and pregnant mice were euthanized on gestational day 18.5 (Supplementary Figure S3A, Supporting Information). The number of implantation sites (including resorption sites, viable births, and non‐viable births) and viable births were recorded for each uterine horn. The placenta and fetus weight were recorded for each viable birth. Viability of the fetus is determined by the morphological integrity and observation of a yolk sac pulsation at the time of harvest. The assessed parameters were normalized to the (undamaged) right uterine horn of each mouse – represented as L/R ratio. The fertility evaluation was tested with 3 unoperated female mice and this group is denoted as the control (CTRL) group.

### Mouse Uterine RNA Extraction

A portion of each uterine horn was collected with 400 µL RNAiso Plus solution (TaKaRa, Japan) in Eppendorf tubes for total RNA extraction. RNA extraction with RNAiso was performed with standard protocol. The tissue was minced and homogenized then vortex for 1 minute for thorough extraction. Chloroform (80 µL, Sigma Aldrich, USA) mixed with the solution and centrifuged at 12 000 rpm for 15 min at 4 °C. The pellet was washed with 75% ethanol twice then air dried. RNA pellet was resuspended in water and the RNA concentrations were measured using the Nanodrop 2000 (Thermo Fisher Scientific) indicated by the 260 and 280 nm absorbance. The RNA samples were stored at −80 °C until use.

### Transcriptomic Analysis

The extracted total RNA was processed for RNA bulk sequencing at Beijing Genomics Institute (BGI). Enrichment of mRNA was performed on total RNA using oligo(dT)‐attached magnetic beads. The enriched mRNA with poly(A) tails was fragmented using a fragmentation buffer, followed by reverse transcription using random N6 primers to synthesize cDNA double strands. The synthesized double strand DNA was end‐repaired and 5’‐phosphorylated with a protruding ‘A’ at the 3’end forming a blunt end, followed by ligation of a bubble‐shaped adapter with a protruding ‘T’ at the 3’end. The ligation products were PCR amplified using specific primers. The PCR products were denatured to single strand, and then single‐stranded circular DNA libraries were generated using a bridged primer. The constructed libraries were quality‐checked and sequenced after passing the quality control. The raw reads after passing quality control were filtered with SOAPnuke (Version 1.5.6) and aligned to the reference sequences (HISAT2, Version 2.2.1). Afterwards, the gene expression level analysis was performed (RSEM, version 1.3.1). DEGs between samples were assessed with DESeq2 (version 1.4.5). DEGs with a cut‐off of Q value < 0.05 or FDR < 0.001 were selected for further evaluation. Data cleaning was performed using R in the RStudio (R Development Core Team, 2023; RStudio Team, 2023). DEGs of interests were visualized with Venn diagram. Gene Ontology functional enrichment analysis was performed with g: Profiler.

### Statistical and Image Analysis

All data analysis and graphics were produced with GraphPad Prism software (Version 8.3.1, GraphPad Software Inc., USA). Data on graph are displayed as mean ± SEM. Sample size for each experiment are indicated in the captions of the corresponding figures. Distribution normality was tested using the Shapiro–Wilk test. Two group comparisons were tested with unpaired Student t‐test for parametric or Mann‐Whitney U test for non‐parametric. Three or more groups were compared with the ordinary one‐way ANOVA followed by Tukey's test was used for parametric data or Kruskal‐Wallis test followed by Dunn's post‐hoc test for non‐parametric data. A p‐value less than 0.05 is considered statistically significant.

Digital images were obtained by camera imaging or microscope imaging system as described in each experimental section. Image manipulation and data quantification was performed with the Image J software built‐in function or plug‐in (National Institutes of Health, USA) unless specified otherwise.

### Ethics Approval Statement and Patient Consent Statement

All clinical samples were collected after obtaining written informed consent from the patients. The sample collection and human subject involvement in this study has been approved by the Institutional Review (UW20‐465) and The Institutional Review Board of The University of Hong Kong Shenzhen Hospital ([2018]94).

Experimental procedures involving the use of animals have been approved by the Committee on Use of Live Animals in Teaching and Research, The University of Hong Kong (CULATR No. 5532‐20 and 6002–22). License to conduct animal experiments were obtained from Department of Health, the Government of the Hong Kong Special Administrative Region prior to any hands‐on practice with animal. The housing of animals was carried out under an AAALAC International accredited program at the Centre for Comparative Medicine Research, The University of Hong Kong under Specific Pathogen Free (SPF) conditions.

## Conflict of Interest

The authors declare no conflict of interest.

## Author Contributions

J.X. performed data curation, formal analysis, investigation, methodology, validation, wrote original draft, wrote, reviewed, and edited the manuscript; P.C. performed conceptualization, supervision, wrote, reviewed draft; E.N. performed funding acquisition, methodology, resources, wrote and reviewed the draft; S.H. performed methodology, validation, resources, wrote and reviewed the manuscript; Z.Y. performed methodology, resources, wrote and reviewed the manuscript; L.X. performed methodology, resources, wrote and reviewed the manuscript; L.M. performed conceptualization, funding acquisition supervision, visualization, wrote and reviewed the manuscript; S.Z. performed funding acquisition, methodology, resources, wrote and reviewed the manuscript; W.Y. performed conceptualization, funding acquisition, supervision, visualization, wrote, reviewed, and edited the manuscript; R.C. performed conceptualization, funding acquisition, methodology, supervision, wrote, reviewed, and edited the manuscript.

## Supporting information



Supporting Information

## Data Availability

The data that support the findings of this study are available from the corresponding author upon reasonable request.
