# Supplementary material for: Human Endometrial Extracellular Matrix Hydrogel Facilitated Endometrial Mesenchymal Stem Cells for Endometrial Regeneration
Source: Adv Healthc Mater. 2025 Sep 21;15(3):e01767. doi: 10.1002/adhm.202501767 (PMC12817108; doi:10.1002/adhm.202501767)

**SUPPORTING INFORMATION**

**Human endometrial extracellular matrix hydrogel facilitated endometrial mesenchymal stem cells for endometrial regeneration**

*Jingwen Xu^1^, Philip C N Chiu^1,2^, Ernest H Y Ng^1,2^, Sentao Hu^3,4^, Zi Ye^3,4^, Liaobing Xin^4^, Lie Ma^3,4^, Songying Zhang^4^, William S B Yeung^2,1^, Rachel W S Chan^1,2^*


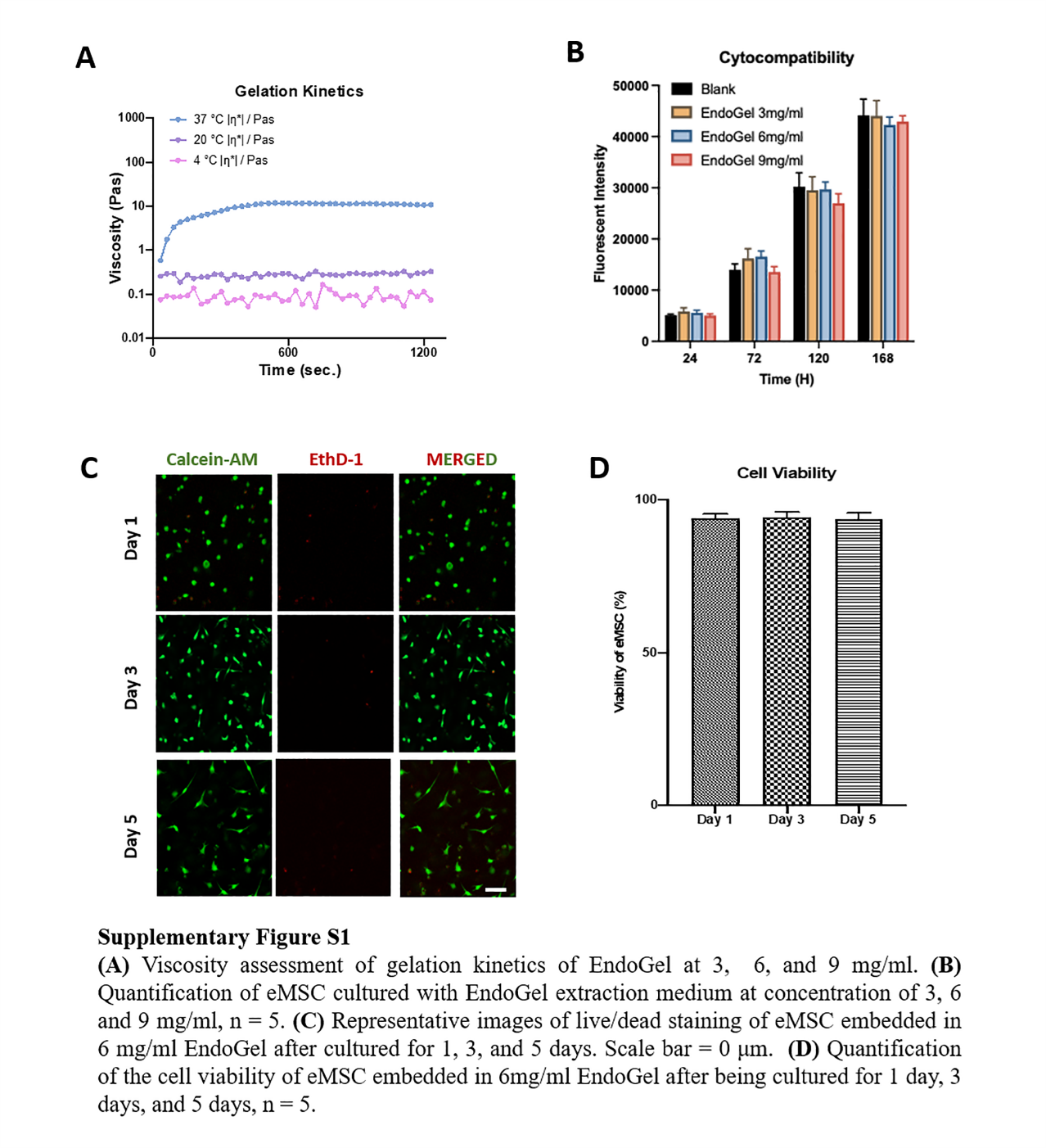


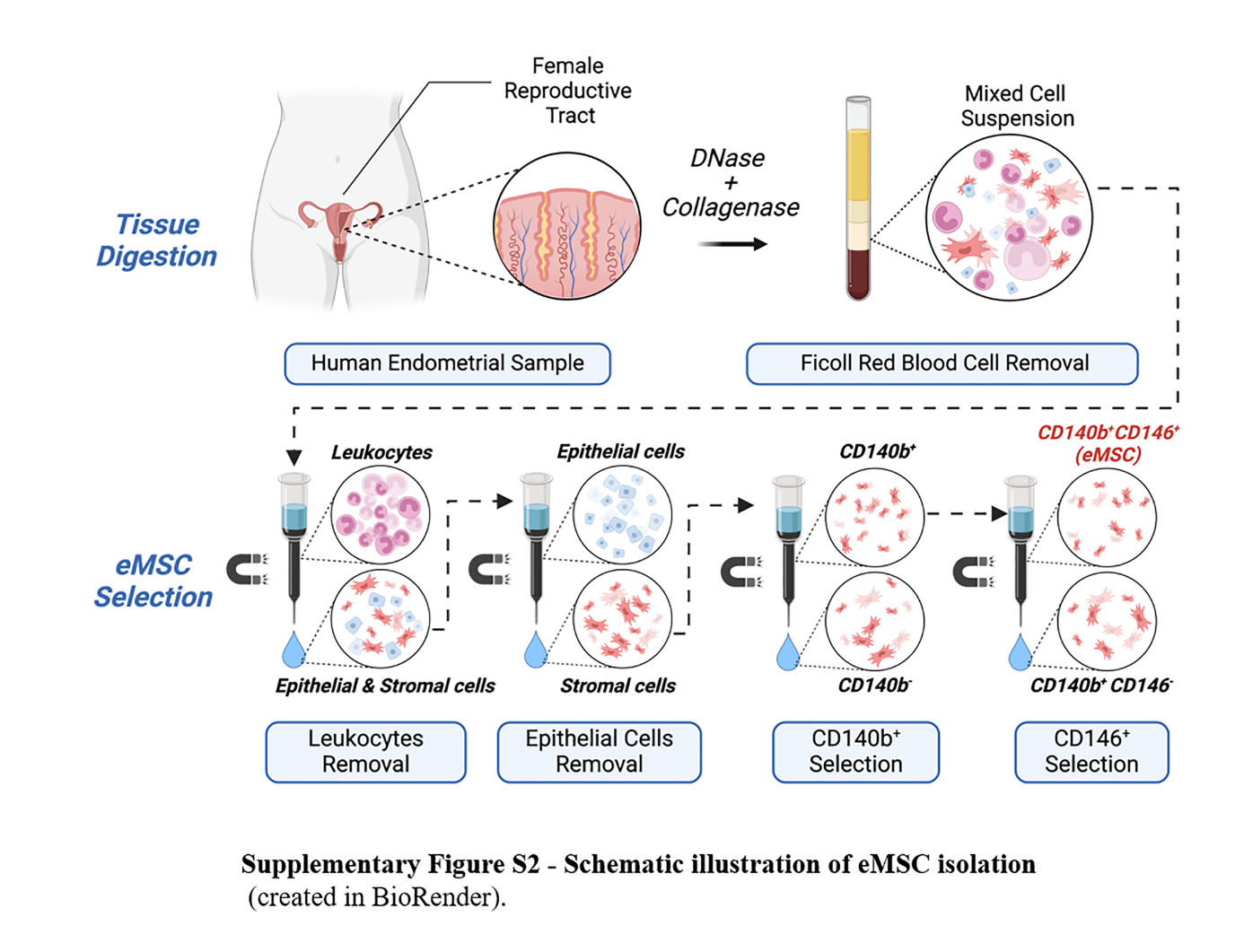


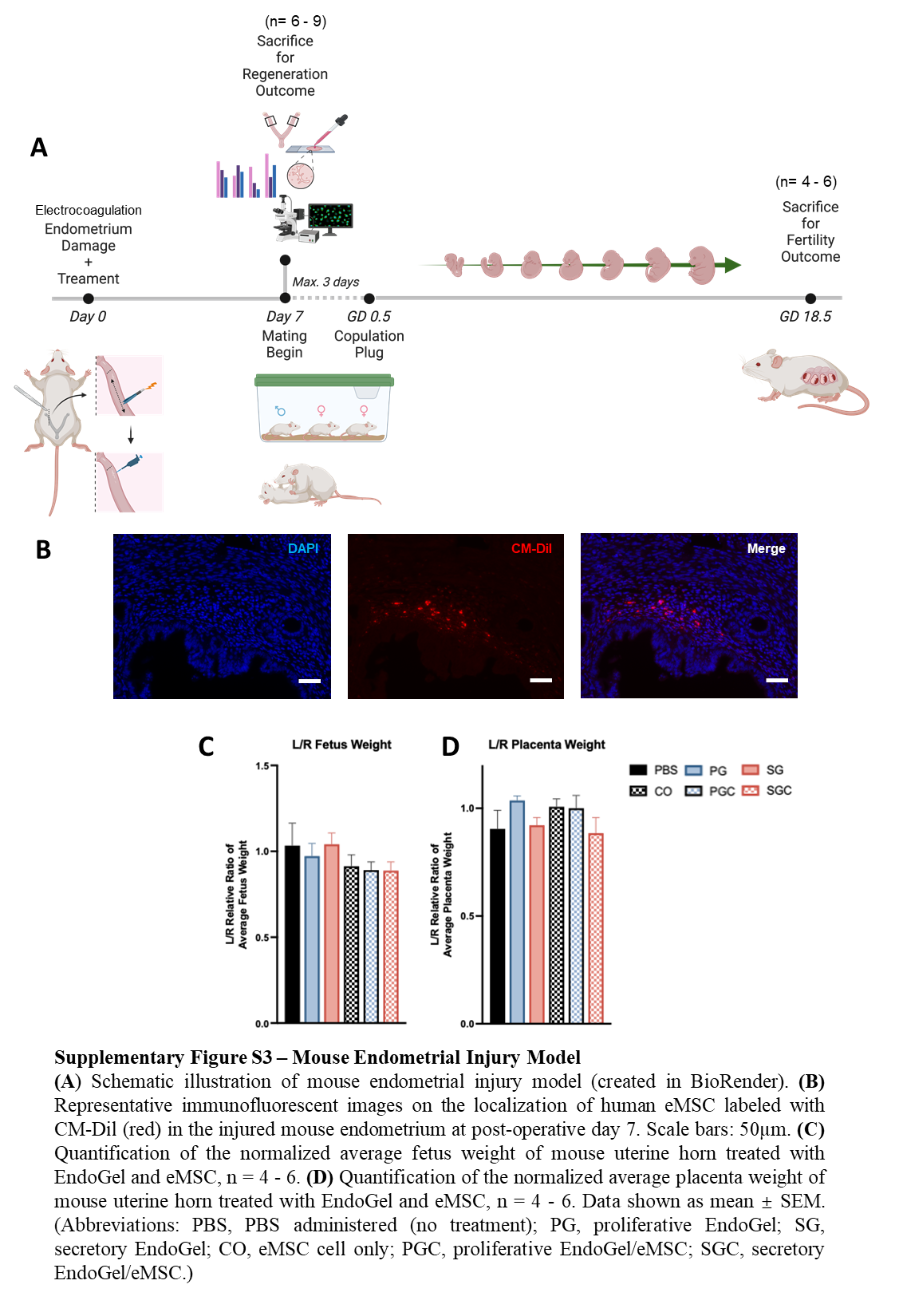


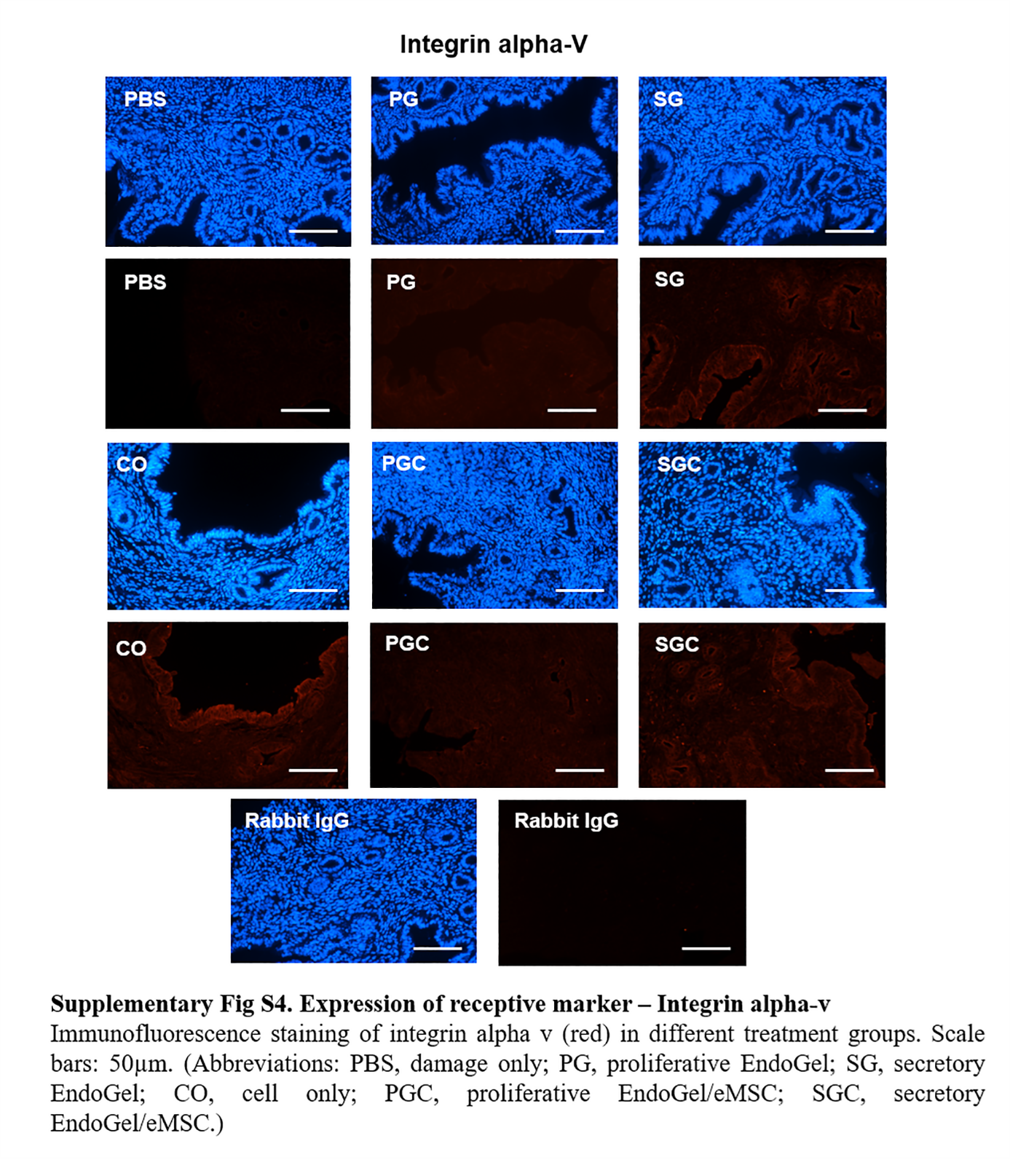


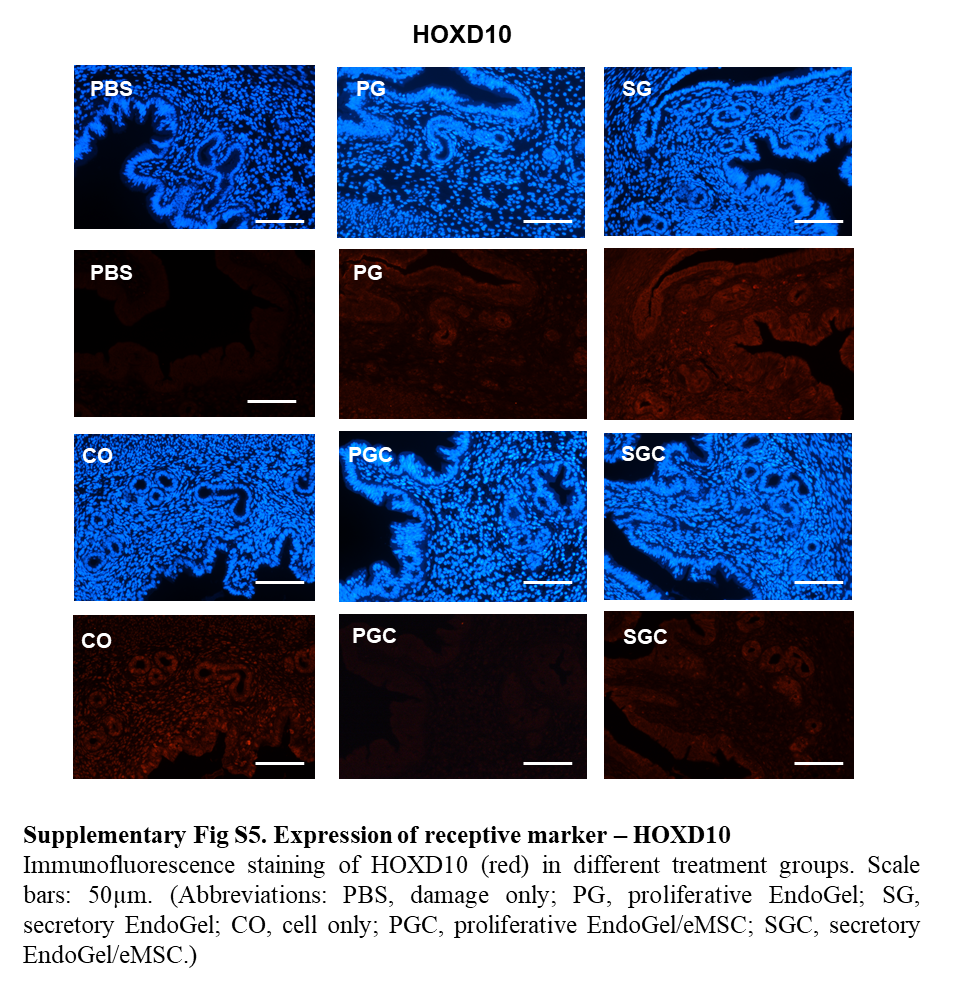

Supplement: Supplementary file 1 — Supporting Information [file ADHM-15-0-s001.docx]
